# Localization of heme biosynthesis in the diatom *Phaeodactylum tricornutum* and differential expression of multi-copy enzymes

**DOI:** 10.3389/fpls.2025.1537037

**Published:** 2025-03-04

**Authors:** Shun-Min Yang, Ansgar Gruber, Kateřina Jiroutová, Jitka Richtová, Marie Vancová, Martina Tesařová, Petra Masařová, Richard G. Dorrell, Miroslav Oborník

**Affiliations:** ^1^ Institute of Parasitology, Biology Centre Czech Academy of Sciences (CAS), České Budějovice, Czechia; ^2^ Faculty of Science, University of South Bohemia, České Budějovice, Czechia; ^3^ Department of Computational, Quantitative and Synthetic Biology (CQSB, UMR7238), Institut de Biologie Paris-Seine (IBPS), Centre National de la Recherche Scientifique (CNRS), INSERM, Sorbonne Université, Paris, France

**Keywords:** chloroplast, tetrapyrrole, horizontal gene transfer, algae, organelle, evolution, endosymbiosis

## Abstract

Heme is essential for all organisms. The composition and location of the pathway for heme biosynthesis, have been influenced by past endosymbiotic events and organelle evolution in eukaryotes. Endosymbioses led to temporary redundancy of the enzymes and the genes involved. Genes were transferred to the nucleus from different endosymbiotic partners, and their multiple copies were either lost or retained, resulting in a mosaic pathway. This mosaic is particularly complex in organisms with eukaryote-derived plastids, such as diatoms. The plastids of diatoms are clearly derived from red algae. However, it is not entirely clear whether they were acquired directly from a red algal ancestor or indirectly in higher-order endosymbioses. In the diatom *Phaeodactylum tricornutum*, most enzymes of the pathway are present in a single copy, but three, glutamyl-tRNA synthetase (GluRS), uroporphyrinogen decarboxylase (UROD) and coproporphyrinogen oxidase (CPOX), are encoded in multiple copies. These are not direct paralogs resulting from gene duplication within the lineage but were acquired horizontally during the plastid endosymbioses. While some iso-enzymes originate from the host cell, others originate either from the genome of the cyanobacterial ancestor of all plastids or from the nuclear genome of the eukaryotic ancestor of the diatom complex plastid, a rhodophyte or an alga containing rhodophyte-derived plastids, a situation known as pseudoparalogy. Using green fluorescent protein-tagged expression and immunogold labeling, we experimentally localized all enzymes of the pathway in *P. tricornutum*, and confirmed their localization in the plastid, with a few possible exceptions. Our meta-analyses of transcription data showed that the pseudoparalogs are differentially expressed in response to nitrate starvation, blue light, high light, high CO_2_, and the cell cycle. Taken together, our findings emphasize that the evolution of complex plastids via endosymbiosis has a direct impact not only on the genetics but also on the physiology of resulting organisms.

## 1 Introduction

Heme is an iron-coordinated tetrapyrrole that is essential for life. It contributes to electron transport cascades in photosystems and respiratory chains, acts as a cofactor in many enzymes and is an important signaling and regulatory molecule (Durham and Millett, 2005; Mense and Zhang, 2006; Zámocký et al., 2014). Unlike eukaryotic heterotrophs, which synthesize the first common precursor for heme biosynthesis, aminolevulinic acid (ALA), by the condensation of glycine and succinyl-CoA catalyzed by ALA synthase (C4 pathway), ALA is synthesized by most eukaryotic phototrophs from glutamate (glutamyl-tRNA) (C5 pathway). In this pathway, which is specific for bacteria (except Alphaproteobacteria) and most eukaryotic phototrophs (except chromerids), ALA is formed in three steps catalyzed by glutamyl-tRNA synthetase (GluRS), glutamyl-tRNA reductase (GluTR) and glutamate-1-semialdehyde aminotransferase (GSA-AT) (Beale et al., 1975; Oborník and Green, 2005; Cihlář et al., 2019; Kořený et al., 2022). The rest of the heme pathway is the same in all eukaryotes: 1) Three steps catalyzed by aminolevulinic acid dehydratase (ALAD), porphobilinogen deaminase (PBGD) and uroporphyrinogen III synthase (UROS) form the first cyclic tetrapyrrole, uroporphyrinogen III. 2) The other three steps are catalyzed by uroporphyrinogen III decarboxylase (UROD), coproporphyrinogen III oxidase (CPOX) and protoporphyrinogen III oxidase (PPOX) (Layer et al., 2010) and yield protoporphyrin IX. Alternatively, oxygen-independent CPOX (CPOX-ind) is also found in most bacteria and eukaryotes. The pathway ends with the final iron chelation of the porphyrin macrocycle by ferrochelatase (FeCH) to produce heme. Alternatively, also chlorophyll can be synthesized from protoporphyrin IX by seven other enzymes, starting with the incorporation of magnesium by Mg chelatase (MgCH). In photosynthetic eukaryotes, heme biosynthesis is strictly compartmentalized and tightly regulated, as the intermediate porphyrins are photosensitizers and can generate photooxidative stress and damage the cell (Brzezowski et al., 2015). The entire heme pathway is localized in the plastid, with most enzymes being of plastidic/cyanobacterial origin (Oborník and Green, 2005; Cihlář et al., 2016; Kořený et al., 2022). The pathway starts with loaded glutamyl tRNA. While the tRNA is encoded on the plastid genome (Oudot-Le Secq et al., 2007), the glutamyl-tRNA synthetase catalyzing this step is nucleus-encoded, like all other enzymes of the pathway. Some of the enzymes are present in multiple copies in the nuclear genomes of algae and plants (Oborník and Green, 2005; Cihlář et al., 2016). Such multiple copies of genes can arise not only by duplication (leading to paralogous genes) but also by the transfer of homologous genes from the endosymbiont (Keeling and Archibald, 2008), such multiple copies are referred to as pseudoparalogous genes (Koonin, 2005). The phylogenetic affinity of the heme biosynthesis proteins may therefore serve to some extent as an endosymbiotic marker.

Diatoms are marine and freshwater algae with rhodophyte-derived complex plastids. They belong to the ochrophytes, the photosynthetic crown group of Stramenopiles. Diatoms contribute immensely to global photosynthetic carbon fixation, as they are responsible for up to 40% of primary production in the ocean (Nelson et al., 1995; Dugdale and Wilkerson, 1998; Gruber and Oborník, 2024). The genes involved in heme biosynthesis in diatoms were first investigated by phylogenetic analysis and prediction of protein subcellular localization (Oborník and Green, 2005) using genome sequence data from the centric diatom *Thalassiosira pseudonana* (Armbrust et al., 2004). These and later analyses with the pennate species *Phaeodactylum tricornutum* showed that most diatom heme biosynthesis enzymes are encoded by single-copy genes, with the exception of glutamyl-tRNA synthase (GluRS), uroporphyrinogen decarboxylase (UROD) and coproporphyrinogen oxidase (CPOX). These are present in multiple copies, derived either from bacteria, from the nucleus of the eukaryotic ancestor of diatom plastids, or from the cyanobacterial ancestor of all plastids (Oborník and Green, 2005; Cihlář et al., 2016, 2019; Sharaf et al., 2019). Similar rearrangements of intracellular locations and paralogs/pseudoparalogs of genes of different phylogenetic origin are also found in other groups of algae with primary or complex plastids (Kořený and Oborník, 2011; Kořený et al., 2013, 2022; Cihlář et al., 2016, 2019; Matsuo and Inagaki, 2018; Richtová et al., 2021; Gruber and Oborník, 2024).

Comparative work on enzyme localization between various organisms with complex plastids of red algal origin showed that in the case of heme pathway enzymes, careful experimental verification of predictions is required and that results cannot be easily transferred between organisms (Richtová et al., 2021). Annotations and targeting predictions of the intracellular distribution of heme biosynthesis in diatoms in previous studies have focused on the centric diatom *Thalassiosira pseudonana* (Oborník and Green, 2005), and may have been challenged by the difficulty of gene modelling in the absence of transcript coverage in the early sequencing projects, and by the unavailability of specialized bioinformatics tools for targeting predictions at that time. Therefore, we used updated gene models and the latest prediction programs, combined with experimental localization using genetic transformations and immuno-electron microscopy to elucidate the detailed localization of the heme pathway enzymes in *P. tricornutum.* Furthermore, we investigated the role of the multicopy genes for UROD and CPOX, in order to find out if they are functionally redundant, or specialized adaptations.

## 2 Materials and methods

### 2.1 Heme pathway genes

All gene sequences were downloaded from NCBI, EnsemblProtists (https://protists.ensembl.org/Phaeodactylum_tricornutum/Info/Index), EST database (https://www.diatomics.bio.ens.psl.eu/EST3/est3.php) (Filloramo et al., 2021)(https://borealisdata.ca/file.xhtml?fileId=159710&version=1.0), and (Giguere et al., 2022) (https://github.com/dgiguer/phaeodactylum-tricornutum-genome). The heme biosynthetic pathway genes in *P. tricornutum* were first acquired from the KEGG database (https://www.genome.jp/pathway/pti00860). For each gene, the NCBI-GeneID was retrieved, and protein sequences were downloaded from NCBI. Each protein sequence was used as the query for tblastn search (expected threshold 0.05) in NCBI to find all homologous genes. *UROD2* was found in this search. The incomplete sequence of *UROD3* in NCBI (XP_002184319.1) was found complete in the JGI database (https://mycocosm.jgi.doe.gov/cgi-bin/dispGeneModel?db=Phatr2&fTable=JAM_UserModels&fId=379), and named “*UROD3_56673*” in this study. *CPOX-ind* was detected by tblastn (expected threshold 0.05) in NCBI using a protein sequence of oxygen-independent coproporphyrinogen III oxidase from *Escherichia coli* (GAB0561990.1). The above coding sequences of heme pathway enzymes were used to blast search the two new long-read genomes (Filloramo et al., 2021; Giguere et al., 2022) in the Geneious Prime Software (Dotmatics) with the Megablast program (max E-value 0.05). The same coding sequences were used for searching the version 3 annotation in EnsemblProtists (https://protists.ensembl.org/Multi/Tools/Blast) with blastn and normal sensitivity. The gene names and gene IDs were listed in **Table 1**. The subcellular localizations of all the enzymes were predicted by ASAFind 2.0 (Gruber et al., 2023), which is based on TargetP 2.0 (Almagro Armenteros et al., 2019a), and by HECTAR v1.3 (Gschloessl et al., 2008). All possible 5’ upstream start codons of heme pathway genes were examined in their genomic sequences until a stop codon appeared, and the transcription of the probable upstream presequences was analyzed by blanstn using the coding sequence as query against the EST database (https://www.diatomics.bio.ens.psl.eu/EST3/blast.html) (Maheswari et al., 2005), and RT-PCR (primers listed in **Supplementary Table S1**) was used to amplify the transcripts.

**Table 1:** *In silico* prediction of heme pathway enzyme localizations in *P. tricornutum*.

			Prediction of subcellular localization	
Gene	Phatr3 Gene IDs	Variants	ASAFind 2.0	HECTAR v1.3
GluRS1	Phatr3_J51430		noTP*	other localization
GluRS2	Phatr3_EG02218		mTP*	chloroplast
GluTR	Phatr3_J54134		Plastid, high	Signal peptide
GSA-AT	Phatr3_J36347		Plastid, low	chloroplast
ALAD	Phatr3_J41746	1.1	Plastid, high	chloroplast
		1.2	Plastid, high	chloroplast
PBGD	HEMC Phatr3_J51811		Plastid, high	chloroplast
UROS	HEMD Phatr3_J44610	1.1	Plastid, low	Signal peptide
		1.2	Plastid, high	chloroplast
UROD1	HEME_2 Phatr3_J19188		Plastid, high	chloroplast
UROD2	HEME_1 Phatr3_J20757	2.1	Plastid, low	chloroplast
		2.2	Plastid, low	chloroplast
UROD3	HEME Phatr3_J16140		mTP*	chloroplast
CPOX1	HEMF_2 Phatr3_J10640		Plastid, high	Signal peptide
CPOX2	HEMF_3 Phatr3_J12186		PPC	chloroplast
CPOX3	HEMF_1 Phatr3_J15068		Plastid, high	chloroplast
CPOX-ind	Phatr3_J51528		Plastid, low	Signal peptide
PPOX	Phatr3_J31109		Plastid, high	chloroplast
FeCH	Phatr3_J26952	1.1	noTP*	Signal peptide
		1.2	noTP*	Signal peptide
		Upstream FeCH	noTP*	other localization

*mTP, mitochondrial transit peptide.

*noTP, no Targeting Peptide, which means no signal peptide and no transit peptide.

### 2.2 Experimental localization of the heme biosynthetic pathway in *Phaeodactylum tricornutum*

#### 2.2.1 Culture of *Phaeodactylum tricornutum*

In general, *P. tricornutum* (strain Pt1) (Martino et al., 2007), obtained from Bigelow NCMA as CCMP632, was cultured in artificial seawater with f/2 nutrients (Guillard, 1975) liquid medium or half concentration of f/2 medium with 1% agar plates at 18°C under 80-200 µmol m^-2^ s^-1^ white light with photoperiod 12/12 day/night in a static incubator unless elsewhere mentioned.

#### 2.2.2 Transformation vector *PtNR-GFP*

Nitrate reductase promoter and terminator sequences from the *P. tricornutum* genome (transcript Id:54983, JGI genome database) were amplified by PCR (See **Supplementary Table S1**) and cloned into pBluescript KS+ vector (Stratagene) with *Sac*I, *Not*I, and *Xho*I, *Kpn*I restriction enzyme sites, respectively. To produce a *PtNR-GFP* vector, the marker gene was amplified from the kindly provided *enhanced green fluorescent protein (EGFP)* (FPbase ID: R9NL8) expression vector from Julius Lukeš laboratory (Institute of Parasitology, Biology Centre CAS, Czech Republic) and subcloned into *PtNR* vector with *Hind*III-*Sal*I sites.

#### 2.2.3 Cloning and construct of heme pathway genes

The total RNA of *P. tricornutum* was isolated with a Hybrid-R™ kit (cat. no. 305-101, GeneAll, Korea) according to the manufacturer’s protocol. The RNA was subsequently treated with a Turbo DNA-free™ kit (cat. no. AM1907, Invitrogen™) to remove the DNA contamination. The total RNA then was reverse-transcribed to cDNA with SuperScript IV First-strand synthesis system (cat. no. 18091050, Invitrogen™). The presequences or the whole genes of the heme pathway genes were amplified by PCR (See **Supplementary Table S1**) using Q5 Hot Start High-Fidelity 2X Master Mix (cat. no. M0494S, New England Biolabs). The cloned gene fragments were inserted into the *PtNR-GFP* vector with either restriction enzyme digestion cloning (New England Biolabs) or In-Fusion^®^ Snap Assembly Master Mix (cat. no. 638948, Takara Bio) (see **Supplementary Table S1** for each gene). The PCR products were purified by Monarch^®^ PCR & DNA Cleanup Kit (cat. no. T1030L, New England Biolabs) for restriction enzyme digestion. The digested insert and vector were purified with a Monarch^®^ DNA Gel Extraction Kit (cat. no. T1020L, New England Biolabs) and were ligated by T4 DNA ligase (cat. no. M1801, Promega), respectively. For In-Fusion cloning, the insert and vector were purified with a Monarch^®^ DNA Gel Extraction Kit and assembled as described in the user manual. 5 µL of ligation mixture or 2.5 µL of assembly mixture was used for *E. coli* transformation using One Shot™ TOP10 Chemically Competent *E. coli* (cat. no. C404010, Invitrogen™). The transformed colonies were selected from LB agar plates containing 100 mg/L Carbenicillin and plasmids were extracted by Hybrid-QTM plasmid Rapidprep (cat. no.100-102, GeneAll) with a modified protocol based on (Pronobis et al., 2016). The constructs were confirmed by Sanger sequencing (LightRun tube, Eurofins Genomics) and are shown in **Supplementary Table S1**.

#### 2.2.4 Biolistic transformation of *P. tricornutum*

Two days before the bombardment, 500 mL of a more than 1-month-old densely grown diatom culture was harvested with a 0.2 µm PES filter (cat. no. 83.3941.101, SARSTEDT) and vacuum pump. The cells were rinsed with 30 mL f/2 medium twice and resuspended in 10 mL f/2 medium. 1 mL of resuspended culture was applied onto the center (3.5 cm in diameter) of a half concentration f/2 agar plate (9 cm in diameter) and air-dried until it became a solid lawn. 5 to 8 plates can be made from this procedure. The agar plates were stored in the dark for two days at room temperature.

To prepare the microcarrier, 60 mg of M17 tungsten microparticles (cat. no. 165-2267, Bio-Rad) were vortex washed with 100% ethanol for 2 minutes, 4 times, and then resuspended in 1 mL water. 60 µL of M17 tungsten microparticles were mixed with 2.5 µg *PtNR–transgene-GFP* vector and 2.5 µg zeocin-resistant *pFCPFp-Sh ble* vector (Falciatore et al., 1999). Freshly made 20 µL 0.1 M spermidine (cat. no. S4139, Sigma-Aldrich) and 50 µL 2.5 M CaCl_2_ (cat. no. C2661, Sigma-Aldrich) were quickly added to the DNA-tungsten particles mixture and immediately vortexed for 3 minutes. The particles were pelleted by quick centrifugation for 10 seconds and the supernatant was removed. The particle pellet was washed with 250 µL of 100% ethanol twice and resuspended in 60 µL 100% ethanol. 12 µL of well-mixed particle suspension were added onto the center of each assembled macrocarrier/microcarrier holder and allowed to dry completely.

Biolistic transformation was carried out with PDS-1000/He™ System (Bio-Rad). Rupture discs with 1,550 psi and helium gas were used for bombardment. The diatom agar plate prepared two days ago was placed on the L3 (=9 cm) target shelf. After the transformation, the cells were kept in the dark at room temperature (25 ± 2°C) for two days to recover. Then, each dried pellet was resuspended with 1 mL f/2 medium and spread onto three half concentration of f/2 medium agar plates containing 100 mg/L zeocin for selection. The transgenic colonies became visible 4 to 8 weeks later, and were subcultured for further analysis.

#### 2.2.5 PCR examination of the transgenic diatoms

The transgenic clones were taken from the agar plate via an inoculation loop and mixed with AccuPower^®^ HotStart PCR PreMix (cat. no. K-5051, BIONEER, Korea) containing forward primer (5’-CAGAATTGCCCGGGTGTTCACAA-3’) and reverse primer (5’- GTAGGTCAGGGTGGTCACGA-3’) to amplify the transgenic fragment between the *NR promoter* and *EGFP* gene. The PCR cycler program for the presequence construct was 94°C for 5 minutes followed by 35 cycles of 94°C, 30 seconds denaturation, 54°C, 30 seconds annealing, and 72°C, 1 minute extension. For full-length ALAD, PPOX, and FeCH constructs, the extension at 72°C was performed for 2 minutes. For the GluRS1 and GluRS2 full-length constructs, the extension at 72°C was performed at 3 minutes and 30 seconds. PCRs were terminated with a final extension at 72°C for 5 minutes and stored at 4°C until gel electrophoresis.

#### 2.2.6 Confocal laser scanning microscope

**Figure 1:**
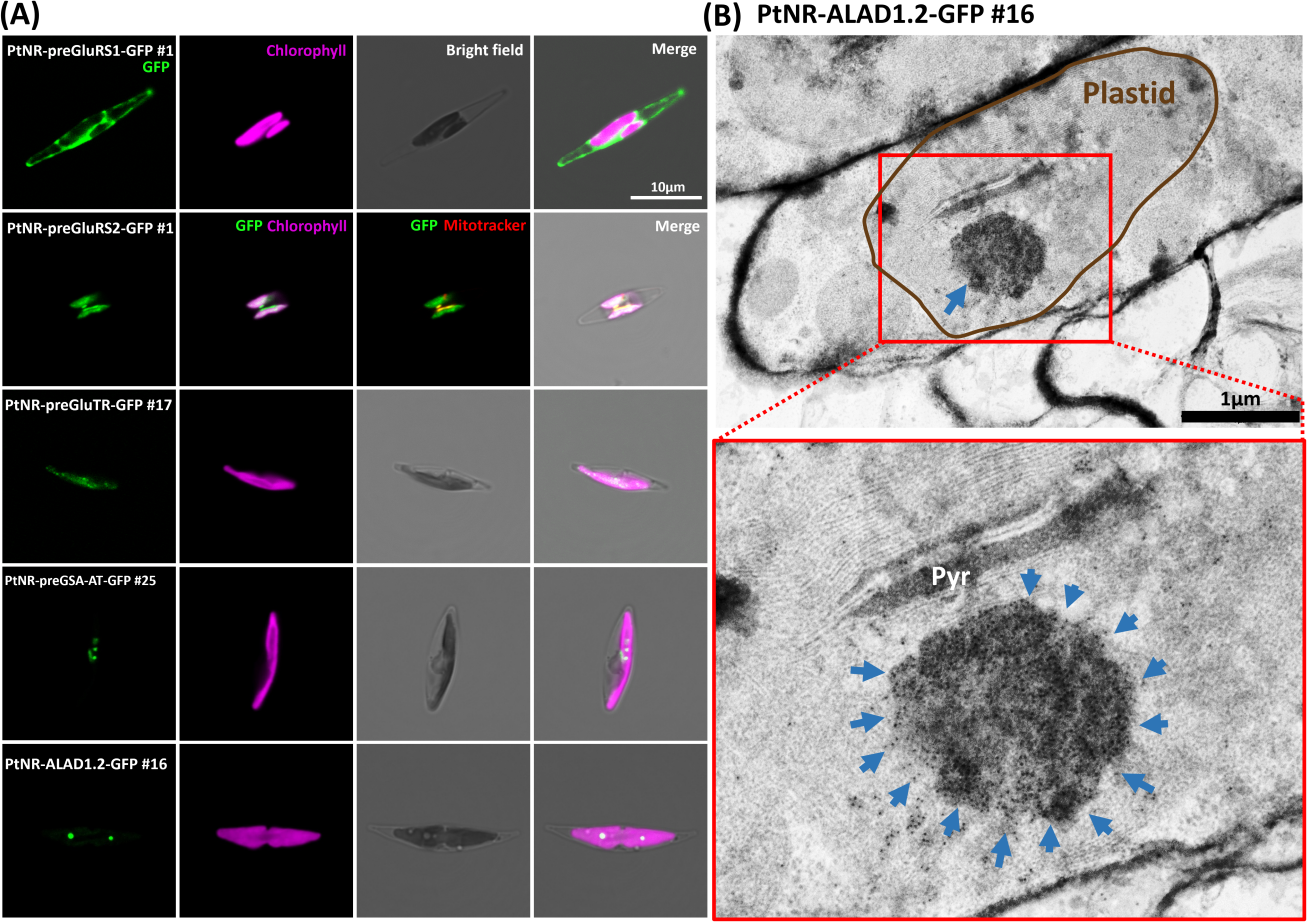
Experimental localization of GluRS1, GluRS2, GluTR, GSA-AT, and ALAD in *P. tricornutum.* **(A)** The confocal images show the localization of the transgenic GFP under the nitrate reductase promoter (*PtNR*). *PtNR-preGluRS1-GFP* clone shows a cytosolic green fluorescence and *PtNR-preGluRS2-GFP* shows plastidial/mitochondrial dual targeting. *PtNR-preGSA-AT-GFP, PtNR-preGluTR-GFP* and *PtNR-ALAD1.2-GFP* show plastidal localization. GFP: Green fluorescent protein signal. Chlorophyll: Chlorophyll autofluorescence. Scale bar = 10 µm. **(B)** Transmission electron microscopy image of *PtNR-ALAD1.2-GFP* clone #16 stained with anti-GFP Immunogold. 10 nm-gold particles target an unknown complex in the plastids, shown magnified in a red rectangle. Pyr, Pyrenoid. Scale bar = 1 µm.

**Figure 2:**
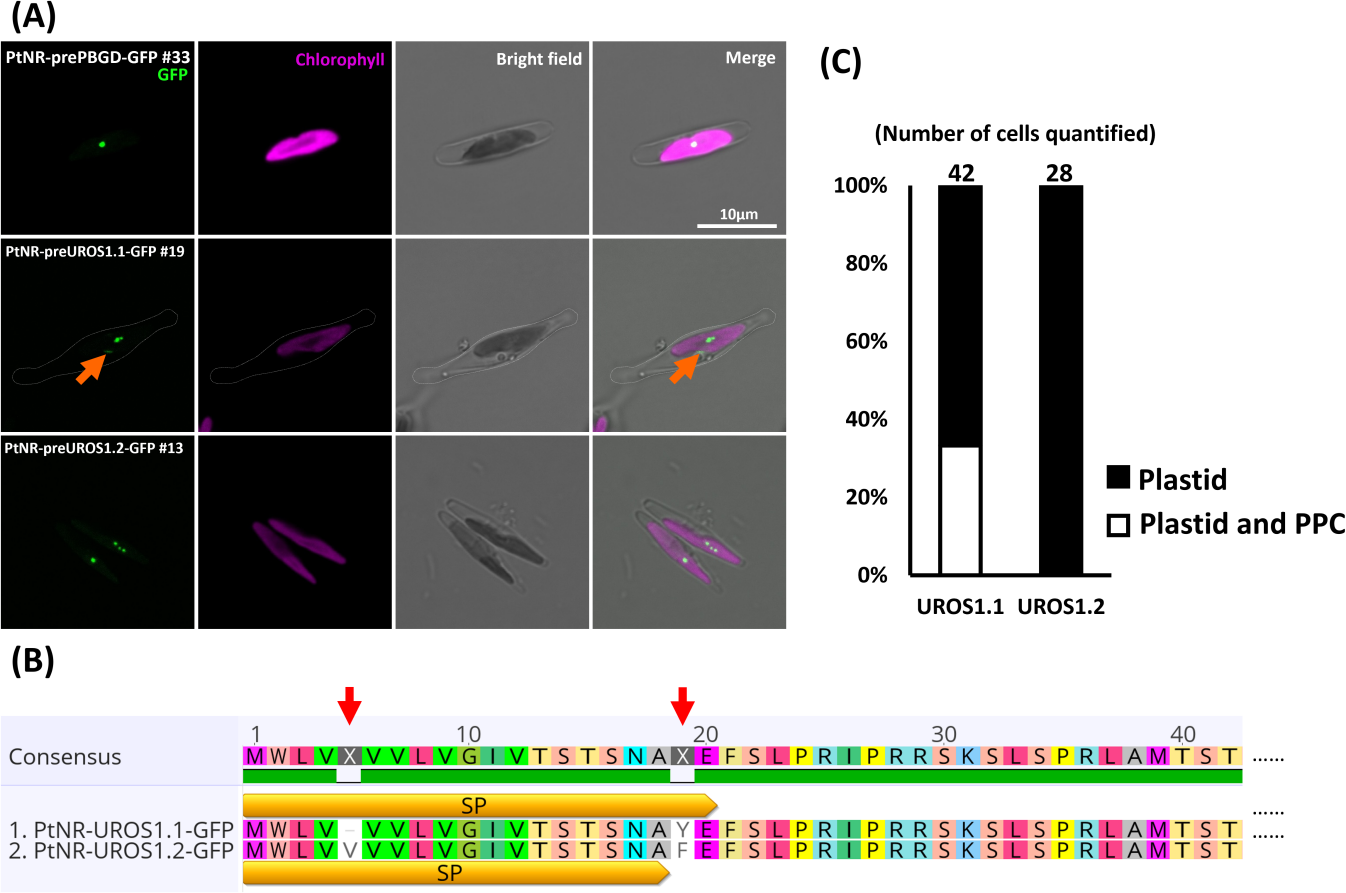
Experimental localization of PBGD and UROS allelic variants in *P. tricornutum.* **(A)** Confocal images show the localization of GFP in transformants with presequence-based constructs activated under the nitrate reductase promoter (*PtNR*). *PtNR-prePBGD-GFP* and *PtNR-preUROS1.2-GFP* show exclusively plastidial localization. *PtNR-preUROS1.1-GFP* shows localization both in a plastidial spot structure and in a probable periplastidial compartment (PPC) (orange arrow). Chlorophyll: Chlorophyll autofluorescence. Scale bar = 10 µm. **(B)** The schematic illustrates the detailed amino acid sequence differences (red arrow) between UROS1.1 and URaS1.2 variants. SP: predicted signal peptide. **(C)** Quantification of UROS1.1 and UROS1.2 localization: 33% of *PtNR-UROS1.1-GFP* cells show plastid and PPC localization and 66% show solely plastidial localization. All PtNR-UROS1.2-GFP cells show plastidial localization.

The transgenic diatom clones were taken from an agar plate and resuspended in 20 µL f/2 medium. Where mitochondrial markers were needed, cells were stained with 500 nM MitoTracker™ Orange CMTMRos (cat. no. M7510, Invitrogen™) in f/2 medium for 20 minutes at room temperature and washed twice with 1 mL f/2 medium. 5 µL culture was applied onto a glass slide and gently covered by a 20 mm x 20 mm coverslip right before microscope observation. Images were acquired using a FV3000 Olympus Confocal Laser Scanning Microscope with a bright field to visualize live cells, and a 488 nm laser (1.0% transmission) to excite GFP and chlorophyll. GFP fluorescence was detected from 500-520 nm while chlorophyll autofluorescence was detected from 670-690 nm. For the Mitotracker staining cells in **Figure 1A**, GFP, chlorophyll autofluorescence and bright field were simultaneously detected as described above. Mitotracker orange was excited by a 561 nm laser (7.5% Laser transmissivity) and the emission was detected from 565-590 nm. The PMT voltage is 350 V for GFP, 470 V for Mitotracker orange, and 450 V for chlorophyll. The Photomultiplier tubes (PMTs) voltage was 410 V for the GFP channel and 350 V for chlorophyll except for UROS1.1 and UROS1.2 in **Figure 2**. For UROS1.1, the PMT voltage was 525 V for GFP and 603 V for chlorophyll; for UROS1.2, the PMT voltage was 480 V for GFP and 549 V for chlorophyll.

The brightness and contrast of all fluorescent images in the main figures were manually adjusted by Lookup Tables (LUTs) in FV31S-SW Viewer software (Olympus) for better visualization. To exclude the crosstalk of chlorophyll autofluorescence, the comparison of the GFP signal between transgenic clones and non-transformed wild-type was equally adjusted in **Supplementary Figure S5**.

#### 2.2.7 Electron microscopy and immunogold labelling

Transgenic *PtNR-ALAD1.2-GFP* diatom clones were grown in liquid f/2 medium without zeocin. The cells were pelleted by centrifugation at 14,000 rpm for 1 minute and high-pressure frozen with 20% BSA in f/2 medium using a Leica EM ICE High-Pressure Freezer. The sample was transferred into a freeze substitution solution containing 0.25% uranyl acetate and 0.01% glutaraldehyde dissolved in 100% acetone at -90°C. After 96 hours, the temperature was gradually increased to -20°C (5°C/h) and samples were incubated for 36h at -20°C before the temperature was raised again to -10°C. The sample was washed three times in 100% acetone and gradually infiltrated by 25%, 50%, and 75% of LR White resin (cat. no. 14381-UC, Electron Microscopy Sciences) diluted in 100% acetone and finally 100% resin at each step for 1 hour. The resin was polymerized by a UV light for 48 hours at -10°C. Ultrathin sections were placed onto formvar-coated nickel grids. For immunogold labelling, grids were blocked in blocking/washing buffer (3% BSA, 0.05% Tween-20, 0.1 M HEPES pH 7.4) for one hour at room temperature and labelled with a 1:40 anti-GFP antibody (ab6556, Abcam) in the same buffer for 15 minutes. After washing 6 times each for 5 minutes with blocking/washing buffer, the samples were incubated in protein-A conjugated to 10 nm gold nanoparticles (CMC Utrecht) for 1 hour at room temperature. The samples were washed 6 times each 5 minutes again and finally rinsed with water. The labelled grids were contrasted with saturated ethanolic uranyl acetate for 30 minutes, rinsed in 30% ethanol and 0.1% lead citrate in approximately 0.1 N NaOH for 20 minutes. The grids were carbon-coated and imaged using transmission electron microscopy (JEM-1400 JEOL) equipped with a Xarosa camera (EMSIS).

### 2.3 *UROD* and *CPOX* expression pattern

RNA-seq data were collected from DiatOmicBase (https://www.diatomicsbase.bio.ens.psl.eu/). Gene expression graphs were downloaded from the RNA-Seq dataset (70 treatments) shown on each gene webpage. Microarray and hierarchical clustering datasets (141 treatments) were collected from Diatom Portal (https://networks.systemsbiology.net/diatom-portal/). Both datasets were used for the correlation calculations in **Supplementary Figure S6B** and **Supplementary Table S2**. The RNA-seq data were used for plotting the differential expression of *UROD* and *CPOX* paralogs. The criteria for filtration were 1) one of the paralogs is up-regulated and the others are downregulated, 2) the highest expression ratio was at least 0.5 higher than the lowest expression ratio in the given RNA-seq dataset, and 3) all adjusted p-values were less than 0.05.

### 2.4 *URODs* and *CPOXs* protein structure and dimer complex prediction

Protein homo-/hetero-dimer complexes were predicted with AlphaFold2.0 (https://colab.research.google.com/github/sokrypton/ColabFold/blob/main/AlphaFold2.ipynb) under default conditions (Jumper et al., 2021; Mirdita et al., 2022). The signal peptide and chloroplast transit peptide were manually truncated before both predictions. Mol* Viewer (Sehnal et al., 2021) was used for the structure visualization.

## 3 Results

### 3.1 Sequence curation and nomenclature of the allelic variants in *Phaeodactylum tricornutum*

Although the *P. tricornutum* genome and transcriptome have been well-studied (Bowler et al., 2008; Ashworth et al., 2016; Rastogi et al., 2018; Ait-Mohamed et al., 2020; Filloramo et al., 2021; Giguere et al., 2022), we found that the sequences encoding GluRS1 (XP_002177350.1), GluRS2 (XP_002184854) and PBGD (XP_002179459.1) from NCBI, and the FeCH (Phatr3_J26952) annotated in *EnsemblProtists*, are incomplete. Therefore, we manually curated all heme pathway genes by comparing datasets from NCBI (version 2 annotation) (Bowler et al., 2008), Ensembl Protists (version 3 annotation) (Rastogi et al., 2018), and two long-read whole-genome sequencing (Filloramo et al., 2021; Giguere et al., 2022) (**Supplementary Figure S1**).

Comparing the NCBI sequences with other sources, we found putative upstream start codons in the genome sequences of *GluRS1*, *GluRS2*, *PBGD*, *UROD1*, *UROD3*, *CPOX1*, *CPOX2*, *CPOX3* and *FeCH*. The upstream start codons of *GluRS1, PBGD, CPOX1 and CPOX3* were fully covered by the EST sequences and the version 3 annotation also correctly identified them. For *GluRS2*, we identified three potential upstream start codons in the version 3 annotation. The gene model (Phatr3_EG02218) starts with”CTG”, and hence does not contain a valid start codon. Therefore, we chose the second upstream ATG as the start codon for the following localization test. In *UROD1*, the upstream coding sequence is not covered by ESTs and we were unable to amplify this sequence by RT-PCR (**Supplementary Figure S2A**). A further blastn search using the upstream 165 bp sequence as query (**Supplementary Figure S1H**) against RNA-seq raw reads of wild-type *P. tricornutum* (NCBI SRA: SRX23012693) showed that the most upstream transcript does not reach the alternative start codon, suggesting that this upstream presequence is unlikely to be transcribed. In the version 3 annotation, *UROD3* has more upstream start codons than in other sequence sources, also spanning an intron (**Supplementary Figure S1J**). Nevertheless, we found that the annotated intron lacked a conserved GU-AG intron splicing motif at both ends, and the EST sequence did not cover this region. Furthermore, we amplified this region by RT-PCR, but the amplicon is larger than expected (400 bp instead of 300 bp) (**Supplementary Figure S2B**). This meant that the annotated intron was not spliced, and we found this sequence was instead a 5’-untranslated region. *CPOX2* also has a possible upstream start codon, but the EST sequences did not cover this region. A blastn search using the 195 bp upstream sequence (**Supplementary Figure S1L**) against RNA-seq raw reads of wild-type yielded several hits covering this region. *FeCH* also has a probable upstream start codon. Although ESTs did not fully cover this region, a putative TATA box motif (TATAGCT) was found 50 bp upstream of the start codon that was not included in the database gene model (**Supplementary Figure S1P**). This sequence could further be amplified by RT-PCR and was detected in blastn searches using the 225 bp upstream sequence (**Supplementary Figure S1P**) against wild-type RNA-seq raw reads (NCBI SRA: SRX23012692). Although the upstream region (hereafter called preFeCH up) appeared to be lowly expressed (based on blastn coverage), we inferred that it is probably still transcribed and chose to test it in parallel in the following localization experiment.

In addition to the putative alternative start codons and miss-annotations of the sequence data, we found several sequence versions in *ALAD*, *UROS*, *UROD2* and *FeCH* when cloning RT-PCR amplicons of the genes. *P. tricornutum* has a diploid genome with 25 pairs of chromosomes (Giguere et al., 2022). The genome sequences among version 2 (Bowler et al., 2008), and two long-read sequencing projects (Filloramo et al., 2021; Giguere et al., 2022) are done using the same strain as we did (Pt1, synonymous name: CCMP2561, CCAP1055/1, CCMP632), and heterozygous allelic variants are known throughout the genome (Yang et al., 2018; Rastogi et al., 2020). In the *ALAD* coding sequences, we found two non-synonymous variations (T112A, cysteine→ serine) and a synonymous variation (G1086T, glycine). UROS has two gene variants with a GTC insertion (encoding valine) at position 10-12, and two non-synonymous variations (A53T, tyrosine → phenylalanine; T887C, valine →alanine). For *UROD2*, we cloned only the 300 bp N-terminal presequence. Within this sequence, we found 6 non-synonymous variations: T40A (methionine → leucine), T50G (isoleucine → serine), A53G (aspartate → serine), A74G (aspartate → serine), G97A (alanine → threonine), T202C (tryptophan → arginine), and one synonymous variation T270C (alanine). In *FeCH*, we found one non-synonymous variation (G1510T, alanine → serine), and 7 synonymous ones (A1167G, alanine; A1224C, threonine; A1326G, leucine; A1335G, leucine; A1392C, leucine; G1434T, alanine; A1461T, isoleucine). The presequence region (340 bp) was however identical in both *FeCH* variants, and therefore only two constructs (the annotated *preFeCH*, and *preFeCH up*, as above), were generated for the localization experiment.

The presequence of *PPOX* was identical in different sequence sources. However, different stop codons were likely present in the complete coding sequence. The NCBI version ends at 1563 bp, the (Giguere et al., 2022) version ends at 852 bp, the (Filloramo et al., 2021) version ended at 873 bp, and the Ensembl Phatr3 annotation crossed another intron and ends at 1602 bp (**Supplementary Figure S1O**) (Rastogi et al., 2018). Our first attempt to amplify the entire coding sequence of the NCBI version by RT-PCR did not match the sequence from the database, as a single thymine was always missing, resulting in a frameshift and slightly elongating the C-terminal end. Comparison with the EST sequence revealed that only thymidine 10 (T_10_) was missing from the transcript. We amplified the full-length coding sequence of the Ensembl Phatr3 version from genomic DNA and found that the T_10_ is edited during maturation of the mRNA (**Supplementary Figure S1O**). We labelled these variants as *ALAD1.1*, *ALAD1.2*, *UROS1.1*, *UROS1.2*, *UROD2.1*, *UROD2.2*, *FeCH1.1* and *FeCH1.2* (**Supplementary Figure S3**). While the single nucleotide polymorphisms (SNPs) in *ALAD*, *UROS*, *UROD2* and *FeCH* indicate the presence of allelic variations in the diploid genome of this strain, we found additional allelic variants in one of the long-read assembled genomes (Filloramo et al., 2021) in *GluRS2, UROD2, UROD3, CPOX3 and PPOX*. The version 3 gene model on the EnsemblProtists displays genetic variation as well (The variant sites per gene can be conveniently browsed in the Ensembl genome portal, here the example of PPOX: https://protists.ensembl.org/Phaeodactylum_tricornutum/Gene/Variation_Gene/Image?db=core;g=Phatr3_J31109;r=29:58300-59974;t=Phatr3_J31109.t1;tl=rs2ymHRTGk85J143-20794965-2049717750).

### 3.2 Targeting prediction of heme biosynthesis enzymes based on the curated sequences

For all curated and cloned presequences or complete gene sequences, we used two independent ochrophyte-specific prediction programs, ASAFind 2.0 (Gruber et al., 2023) and HECTAR v1.3 (Gschloessl et al., 2008) to predict the subcellular localization of the enzymes. These predictors were developed to recognize targeting presequences in the protein sequences of diatoms and other algae with complex plastids. ASAFind 2.0 uses the results of TargetP 2.0 to identify a signal peptide, mitochondrial transit peptide, or absence of these (prediction “other”). When a signal peptide is predicted, ASAFind 2.0 proceeds with scoring of a 25 amino acid sequence window around the signal peptide cleavage site, to further predict plastid or periplastidal compartment (PPC) targeting. For plastid proteins, two confidence levels are distinguished. HECTAR v1.3 uses a combination of several prediction programs and a motif search, which are processed by a multiclass Support Vector Machine to distinguish signal peptides, mitochondrial transit peptides, plastid targeting presequences or the absence of these targeting signals. HECTAR does not distinguish confidence levels for plastid proteins. In *P. tricornutum*, most heme pathway enzymes were predicted as plastid/chloroplast-localized proteins by both algorithms (**Table 1**). A comparison of the two predictors showed slightly different results for GluRS2, GluTR, UROS1.1, CPOX1, CPOX2, and CPOX-ind. HECTAR v1.3 predicted a signal peptide without a plastid targeting signal for UROS1.1 and CPOX-ind, while ASAFind 2.0 predicted the plastid localization (low confidence) of these proteins. For CPOX2, ASAFind 2.0 predicted a PPC localization, while HECTAR v1.3 predicted it to be a plastid protein. Furthermore, the localizations of GluRS2 and UROD3 were assigned to the mitochondrion by TargetP 2.0, while HECTAR v1.3 predicted plastid targeting. FeCHs were not found to possess a recognizable targeting peptide (noTP, namely neither a signal peptide nor a transit peptide) using ASAFind 2.0, but HECTAR predicted a signal peptide for FeCH. Both predictors found no potential targeting presequence in the upstream extended FeCH.

### 3.3 Experimental subcellular localization of the heme pathway in *Phaeodactylum tricornutum*

To experimentally confirm the localization, we fused the N-terminal region (79-111 amino acids) including a presequence of each gene to the fluorescent reporter EGFP (**Supplementary Figure S3B**, **Supplementary Table S1**). Presequence GFP fusion constructs are the current standard for protein localization studies in diatoms, because the presequence itself only minimally interferes with the reporter protein, whereas a full-length fusion may introduce additional challenges to folding, stability and activity of the complete fusion proteins. Furthermore, regulatory mechanisms at the transcriptional or translational level may affect the expression of a full-length fusion construct, making it difficult to detect products of weakly expressed genes. In parallel, we also prepared five full-length coding sequence constructs from *GluRS1*, *GluRS2*, *ALAD1.2*, *PPOX*, and *FeCH1.1*, to confirm the consistency of localization between full-length and presequence constructs, despite the above-mentioned caveats (**Supplementary Figure S3C**, **Supplementary Table S1**). Wild-type *P. tricornutum* strain Pt1 was genetically transformed using the constructs by biolistic bombardment (Falciatore et al., 1999), and transgenic clones were examined by confocal laser microscopy. All of the GFP-positive clones observed with confocal microscopy were further analyzed by PCR to confirm the correctness of the transformation (**Supplementary Figure S4**). Except for *PtNR-CPOX-ind-GFP*, for which only one transgenic clone was obtained, multiple (2 to 14) GFP and PCR-positive clones were analyzed for all constructs (**Table 2**).

**Table 2:** Summary of transgenic clones of all experimental localization.

Gene	Variants	Transgene construct	Number of PCR positive clones	Localization
GFP		*PtNR-GFP*	5	Cytoplasm
GluRS1		*PtNR-preGluRS1-GFP*	5	Cytoplasm
		*PtNR-GluRS1-GFP*	2	Cytoplasm
GluRS2		*PtNR-preGluRS2-GFP*	2	Plastid & Mitochondrion
		*PtNR-GluRS2-GFP*	2	Plastid & Mitochondrion
GluTR		*PtNR-preGluTR-GFP*	3	Plastid
GSA-AT		*PtNR-preGSA-AT-GFP*	11	Plastid
ALAD	1.1	*PtNR-preALAD1.1-GFP*	3	Plastid
	1.2	*PtNR-preALAD1.2-GFP*	5	Plastid
		*PtNR-ALAD1.2-GFP*	2	Plastid
PBGD (HMBS)		*PtNR-prePBGD-GFP*	7	Plastid
UROS	1.1	*PtNR-preUROS1.1-GFP*	3	Plastid & PPC
	1.2	*PtNR-preUROS1.2-GFP*	3	Plastid
UROD1		*PtNR-preUROD1-GFP*	2	Plastid
UROD2	2.1	*PtNR-preUROD2.1-GFP*	3	Plastid
	2.2	*PtNR-preUROD2.1-GFP*	9	Plastid and cytoplasm
UROD3		*PtNR-preUROD3-GFP*	6	Plastid
CPOX1		*PtNR-preCPOX1-GFP*	3	Plastid & PPC
CPOX2		*PtNR-preCPOX2-GFP*	6	Plastid
CPOX3		*PtNR-preCPOX3-GFP (1)*	2	Plastid
		*PtNR-preCPOX3-GFP (2)*	1	Plastid
CPOX-ind		*PtNR-preCPOX-ind-GFP*	1	Plastid
PPOX		*PtNR-prePPOX-GFP*	14	Plastid
		*PtNR-PPOX-GFP*	2	Plastid
FeCH	1.1	*PtNR-preFeCH-GFP*	6	Cytoplasm & Plastid
		*PtNR-FeCH1.1-GFP*	4	Cytoplasm & Plastid
	1.2	N.A	N.A	N.A
Upstream FeCH		*PtNR-preFeCH up-GFP*	4	Cytoplasm

The presequence and full-length *GluRS1* constructs showed cytosolic GFP localization (**Figure 1A**; **Supplementary Figure S5A**), while for *GluRS2* both the presequence and full-length constructs show dual targeting of GFP to plastids and mitochondria (**Figure 1A**; **Supplementary Figure S5B**). The GFP signals for the presequence constructs of *GluTR*, *GSA-AT*, *ALAD1.1*, and *ALAD1.2*, were colocalized with the chlorophyll autofluorescence (**Figures 1A**, **Supplementary Figure S5A**), with some forming intense bright spots. Further examination of detailed localization by transmission electron microscopy and anti-GFP immunogold labelling in cells transformed with *PtNR-ALAD1.2-GFP* (full-length clone #16) revealed an accumulation of gold particles in an inclusion body-like structure in the plastid stroma (**Figure 1B**). The *PBGD* presequence construct also displayed a clear spot signal in the plastid (**Figure 2A**). In the UROS gene variants, a single nucleotide polymorphism between *UROS1.1* and *UROS1.2* (A53T) was located exactly at the conserved signal peptide cleavage site (A↓F), turning the conserved phenylalanine (F) in *UROS1.2* into tyrosine (Y) in *UROS1.1* (**Figure 2B**; **Supplementary Figure S3**). The GFP signal of preUROS1.1-GFP was observed to be localized in the plastid, with some of the signals bound to the plastids, indicating PPC localization (**Figure 2A**), whereas *preUROS1.2-GFP* transformants exhibited exclusively plastidial localization. Further quantification of *preUROS1.1-GFP* cells showed that approximately 33% of the cells have dual PPC- and plastid localization and 66% of the cells have an exclusive plastid localization (**Figure 2C**).

**Figure 3:**
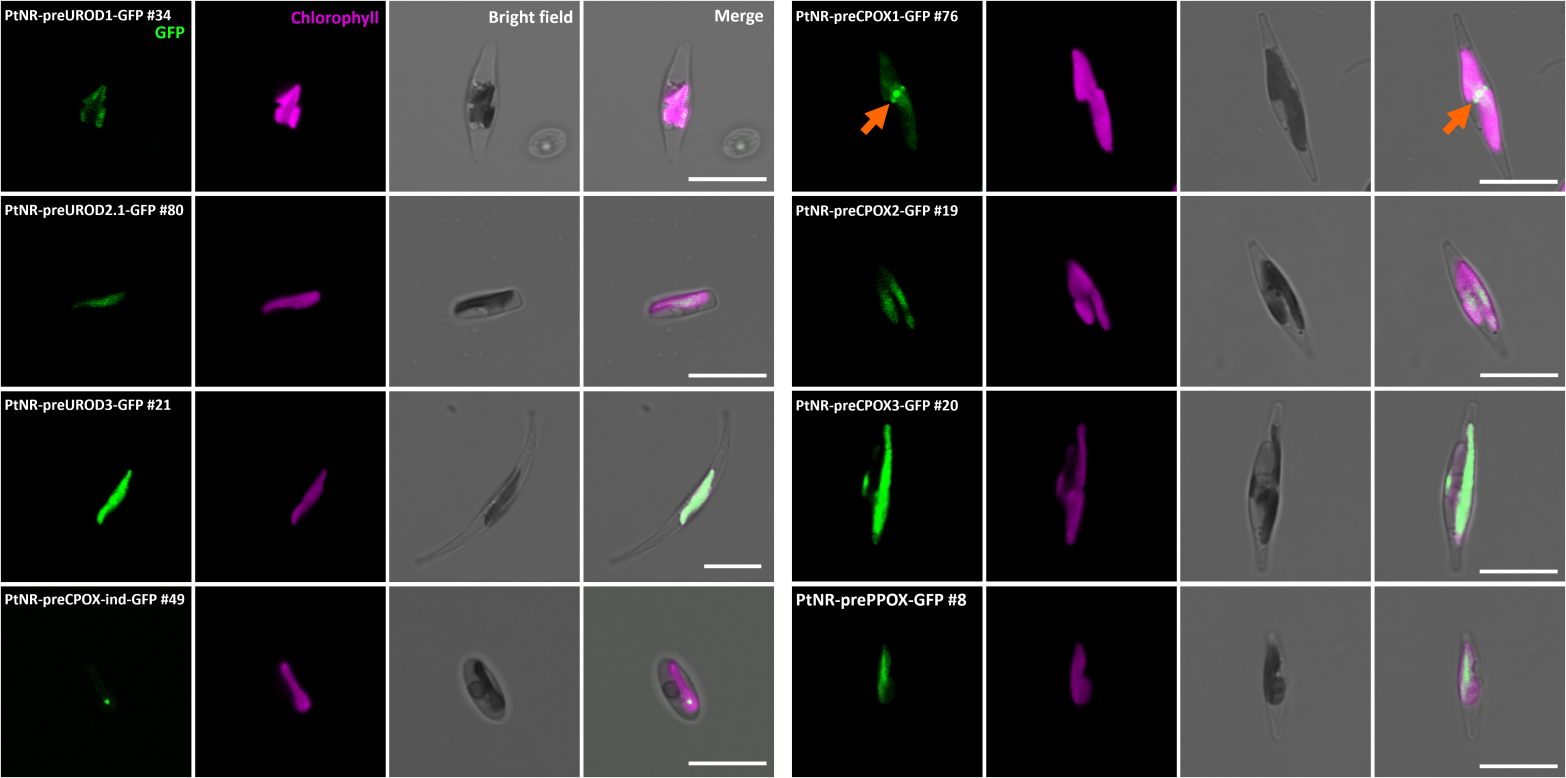
Experimental localization of UROD paralogs, CPOX paralogs, CPOX-oxygen independent (CPOX-ind), and PPOX in *P. tricornutum.* Confocal images show the localization of GFP in transformants with presequence-based constructs activated under the nitrate reductase promoter (*PtNR*). All transformed cell lines show colocalization of GFP signal and chlorophyll. PtNR-preCPOX1-GFP also shows a blob-like GFP signal next to the plastid (orange arrow). GFP: Green fluorescent protein signal. Chl: Chlorophyll autofluorescence. Scale bar = 10 µm.

**Figure 4:**
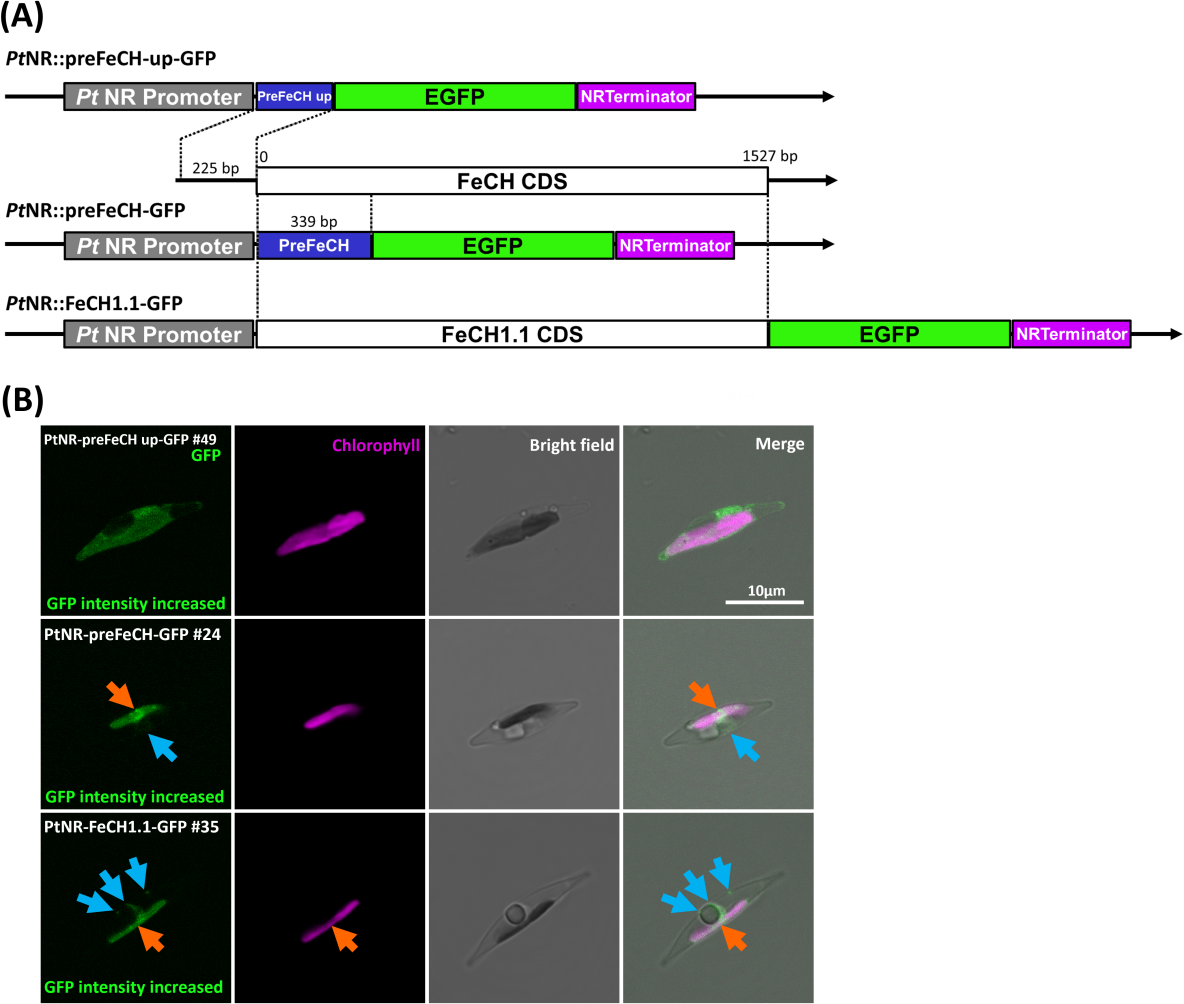
Experimental localization of FeCH in *P. tricornutum.* **(A)** The three different constructs generated for the localization of FeCH. **(B)** Confocal images show the localization of GFP in transformants with presequence-based constructs activated under the nitrate reductase promoter (*PtNR*). The upstream presequence-GFP transformed cell line shows cytosolic localization. FeCH presequence-GFP and full-length *FeCH1.1-GFP* show both plastid and mild cytoplasmic localization. Orange arrows point to GFP signal in the plastid. Blue arrows indicate the cytosolic GFP signal. GFP: Green fluorescent protein signal. Chl: Chlorophyll autofluorescence. BF: Bright field. Scale bar = 10 µm.

The enzymes involved in the next three steps, UROD1, UROD2.1, UROD2.2, UROD3, CPOX1, CPOX2, CPOX3, CPOX-ind (oxygen-independent) and PPOX, were found to colocalize with the chlorophyll signal, indicating localization in the plastid (**Figure 3**; **Supplementary Figure S5**). CPOX1 and CPOX-ind showed a spot-like signal in the plastids. Additionally, the GFP in the *preCPOX1-GFP* transformant showed both a strong spot in the plastid and a blob-like structure, indicating a PPC localization (Gould et al., 2006; Moog et al., 2011) (**Figure 3**; **Supplementary Figure S5C**). Finally, for FeCH, we made three constructs with the genes encoding the enzyme responsible for iron chelation: the *FeCH* presequence (*preFeCH-GFP*), the full *FeCH* coding sequence (*FeCH1.1-GFP*) and the upstream-extended *FeCH* presequence (*preFeCH up-GFP*) (**Figure 4A**). FeCH1.2 had only one amino acid at the C-terminus different from FeCH1.1 and therefore we use FeCH1.1 for full-length gene localization (**Supplementary Figure S3C**). Cell lines transformed with *preFeCH-GFP* and full-length *FeCH1.1-GFP* showed localization both in the plastid and the cytosol. In contrast, the upstream presequence *preFeCH up-GFP* showed a cytosolic localization pattern (**Figure 4B**).

### 3.4 Ferrochelatase may be dually localized in the plastid and cytosol

**Figure 5:**
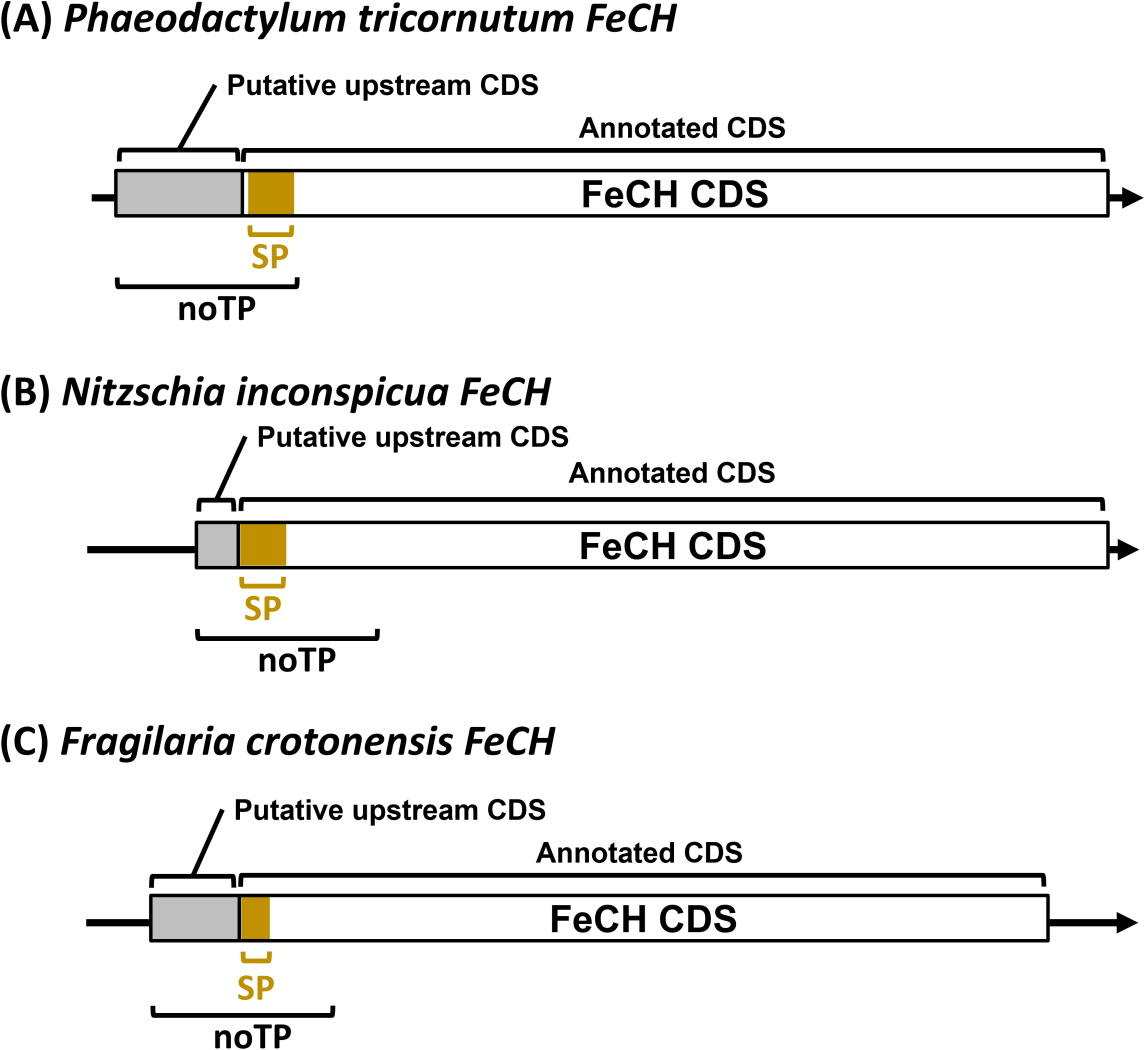
Illustration of the putative upstream alternative start codon in three diatom *FeCH*s that could mask the targeting presequence. A schematic illustrates the annotated and putative upstream coding sequence FeCH in **(A)** *P. tricornutum*, **(B)** *N. inconspicua* and **(C)** *F. crotonensis*. The grey box is the putative upstream coding sequence (CDS). The brown color in the annotated CDS is a predicted signal peptide (SP). noTP: no transit peptide predicted.

Since the upstream alternative start codon could alter localization, we also examined the FeCH genes in other diatoms, including the centric diatoms *Thalassiosira pseudonana* and *Chaetoceros tenuissimus*, the raphid pennate diatoms *Fistulifera solaris*, *Fragilariopsis cylindrus*, *Nitzschia inconspicua*, *Pseudo-nitzschia multistriata* and *Seminavis robusta* as well as the araphid pennate diatom *Fragilaria crotonensis*. In these diatoms, we observed the putative alternative start codon upstream of the annotated gene model in the genome sequences of *N. inconspicua* (FeCH NCBI: KAG7366225.1) and *F. crotonesis* (FeCH NCBI: KAI2498131.1) (**Figure 5**). Using TargetP2.0, these extended sequences were able to change the predicted localization from signal peptide (likely plastid targeting) to a different localization (cytosolic), as we observed in *P. tricornutum* (**Figure 4**).

### 3.5 *UROD* and *CPOX* paralogs and pseudoparalogs in *P. tricornutum* show different transcript abundances under various environmental conditions

Since the proteins encoded by the *P. tricornutum UROD* and *CPOX* pseudoparalogs were all localized in the plastid despite their evolutionarily mosaic origin, we further investigated their expression using various transcriptomic data to determine whether specific conditions differentially regulate their transcription. For this purpose, we used a pre-calculated RNA-seq dataset (DiatOmicBase) (Villar et al., 2024) that includes different nutrient limitations, light colors, intensity, photoperiod changes, temperature changes, an increase of CO_2_ and pH, the presence of pollutants, cell cycle stages, and different cell morphologies. Among the 70 treatments, we found significant differences in the expression of *UROD* isoforms in only four conditions, and *CPOX* isoforms in seven conditions (**Supplementary Figure S6A**). These conditions included nitrate starvation, aureochrome (diatom-specific blue light receptor) knockout mutants, cell cycle, high light and high CO_2_ levels (Matthijs et al., 2017; McCarthy et al., 2017; Mann et al., 2020; Agarwal et al., 2021). Furthermore, by combining the expression profile from RNA-seq (DiatOmicBase) (Ait-Mohamed et al., 2020; Villar et al., 2024) and a Microarray dataset (Diatom Portal v1) (Ashworth et al., 2016), we calculated the correlation coefficient between the gene expression of *URODs* and *CPOXs* and the expression of other genes involved in the biosynthesis of heme and chlorophyll (**Supplementary Figure S6B**, **Supplementary Table S2**). The results showed that *UROD2*, *UROD3* and *CPOX3* were highly positively correlated with almost the entire chlorophyll pathway and UROD1, CPOX1 and CPOX2 were median positively correlated, suggesting that these genes are most strongly coregulated with chlorophyll biosynthesis.

## 4 Discussion

### 4.1 The predictions and experimental localizations of the enzymes of heme biosynthesis are in agreement, with the exception of ferrochelatase

Nucleus-encoded plastid-targeted proteins in diatoms and other complex algae possess an N-terminal bipartite targeting sequence consisting of an ER signal peptide followed by a chloroplast transit peptide (Grossman et al., 1990; Bhaya and Grossman, 1991; Lang et al., 1998; Füssy and Oborník, 2018). The predictors HECTAR, and ASAFind (in combination with TargetP 2.0) come to mostly concordant predictions. The lower number of predicted plastid enzymes based on HECTAR is probably due to its lower sensitivity compared to ASAFind (Gruber et al., 2015). The only enzymes with conflicting predictions are GluRS2, UROD3, CPOX2 and FeCH, which we will discuss here. GluRS2 is predicted to be targeted to mitochondria with TargetP 2.0 and plastids with HECTAR v1.3. The experimental results showed dual-targeting and were consistent with both predictions. This could be due to the ambiguous targeting sequence that can be recognized by both types of transport machinery. The result is also consistent with previous work that most ochrophyte aminoacyl-tRNA synthetases (aaRSs) are present in only two isoforms, one cytonuclear and the other dually-targeted to mitochondria and plastids (Gile et al., 2015; Dorrell et al., 2019). Interestingly, a large-scale phylogenomic analysis shows that aaRSs of the non-photosynthetic stramenopile *Actinophyris sol* are solely targeted to the mitochondria of *P. tricornutum*, indicating the ambiguous targeting mechanism has evolved specifically in ochrophytes (Azuma et al., 2022).

ASAFind 2.0 predicted a mitochondrial transit peptide for UROD3; however, when we examined the TargetP 2.0 prediction report, both the mitochondrial transit peptide (probability: 0.4946) and the signal peptide (probability: 0.4634) were of comparable value. An extended search of UROD3 in the other three signal peptide predictors, SignalP 6.0, SignalP 5.0 and SignalP 4.1 (Nielsen, 2017; Almagro Armenteros et al., 2019b; Teufel et al., 2022) revealed a consistent prediction of the signal peptide for this enzyme (probability: SignalP 6.0: 0.9997, SignalP 5.0: 0.5199, SignalP 4.1 mean D: 0.615). This means that this presequence is much more likely to be the ER signal peptide than the mitochondrial transit peptide, which is supported by the fact that we did not observe mitochondrial localization of UROD3 in any of our transgenic experiments.

When comparing the localization of *UROS* and *UROD* variants, we found slight differences in *preUROS1.1-GFP* and *preUROS1.2-GFP* transformants, as well as pre*UROD2.1-GFP* and pre*UROD2.2-GFP* transformants. UROS1.1 and UROS1.2 differ only slightly by a valine deletion and a tyrosine (Y)/phenylalanine (F) substitution. The different amino acids might affect protein transport and signal peptide cleavage.

The *preUROD2.2-GFP* transformants showed strong plastidial and weak cytosolic signals, which is slightly different from the *preUROD2.1-GFP* clones, which showed exclusively plastid localization. The ASAFind 2.0 predictions between the two variants are highly similar and the cleavage sites are almost identical (UROD2.1 - TNA↓WMT; UROD2.2 - TSA↓WMT).

For CPOX2, the signal peptide cleavage site is predicted differently by HECTAR (AAA↓WIP), and TargetP 2.0/ASAFind 2.0 (NGA↓SSN), leading to a PPC prediction of CPOX2 by ASAFind 2.0. However, in the light of our experimental results, the HECTAR prediction seems to be the correct one.

Finally, despite the low probability of a putative plastidial localization of FeCH derived from two predictors (noTP and signal peptide, respectively), both the presequence and the full-length FeCH constructs are localized in the plastid with a low cytosolic signal. This result could be due to the ambiguous targeting sequence or alternative translation, as the prediction of the nearby second downstream start codon shows a high probability for a signal peptide (**Supplementary Figure S7**). The actual translation pattern remains to be clarified. As for the upstream FeCH presequence, the cytosolic localization is consistent with the negative predictions of both predictors.

### 4.2 Ferrochelatase may be dually localized in the plastid and cytosol

In the *preFeCH up-GFP* construct, the presequence has no predictable targeting sequence and we observed weak cytoplasmic localization. Although the upstream sequence region is likely to be weakly transcribed, as only one sequence raw read was found in the BLAST search in the transcriptome raw reads (NCBI SRA: SRX23012692), the extended N-terminal sequence may mask the downstream bipartite targeting sequence and prevent recognition of the signal peptide by the signal recognition particle, which is required for co-translational transport of the nascent protein to the ER lumen as the first step of protein translocation to the plastid. In the haptophyte *Chrysochromulina tobinii* (protein_id: KOO22639.1), the cryptophyte *Baffinella* sp. *CCMP2293* (NCBI: KAJ1472013.1), and the chlorarachniophyte *Bigelowiella natans* (EnsemblProtists ID: e_gw1.1.414.1 and aug1.26_g9356), we found no possible upstream coding alternatives of the *FeCH* presequence. Interestingly, we found an alternative start codon 30 bp upstream of the currently annotated gene model in the cryptophyte *Guillardia theta FeCH* (NCBI: XP_005839672.1). This extended sequence is predicted by TargetP 2.0 to be the mitochondrial transit peptide (Cihlář et al., 2016). This upstream sequence is most likely a true start codon, as we also found this sequence in an RNA-seq raw read (NCBI SRA ID: SRX549023), suggesting its transcription, expression and localization in the mitochondrion.

Experimental validation in animals shows that FeCH and PPOX form a complex with other related proteins in the mitochondrial membrane (Piel et al., 2019). In mammals, FeCH has also been found in the outer membrane of mitochondria, where it is involved in the reverse removal of iron from porphyrin (Sakaino et al., 2009). In green algae, FeCH and PPOX are associated together with the chloroplast membrane (van Lis et al., 2005). In the red alga *Cyanidioschyzon merolae*, FeCH is not localized in plastids but probably in mitochondria (Watanabe et al., 2013). In land plants, both FeCH isoforms are localized in the chloroplast as well as in the mitochondria of one of the tobacco species and *Arabidopsis* (Chow et al., 1997, 1998; Hey et al., 2016) while in cucumber, both isoforms act exclusively in the plastid (Masuda et al., 2003). Remarkably, a recent study showed that the PPOX of *Arabidopsis* is exclusively located in the chloroplast (Hedtke et al., 2023), suggesting that the mitochondrial FeCH does not form a complex with PPOX. In the non-photosynthetic chrysophyte “*Spumella*” sp. NIES-1846, the FeCH is localized in plastid stroma and in the chrysophyte *Paraphysomonas bandaiensis*, FeCH is localized in PPC (Dorrell et al., 2019). Both ferrochelatases (pseudoparalogs) from the chromerid *Chromera velia* (Cvel_18167, Cvel_26873) were ectopically localized in the PPC of *P. tricornutum* and in the apicoplast of *Toxoplasma* (Richtová et al., 2021), indicating their ability to be targeted to the plastid.

In summary, FeCH is not strictly localized in a specific subcellular compartment but can be localized in different locations in the eukaryotic cell. In many photosynthetic organisms, FeCH contains a chlorophyll a/b binding (CAB) domain at the C-terminal extension (**Supplementary Figure S7**) (Sobotka et al., 2011; Pazderník et al., 2019). Since we found this domain also in *P. tricornutum* FeCH, it is likely that at least one version of FeCH is localized in the plastid. In addition, FeCH and PPOX tend to form an enzymatic complex and should therefore be present in the same compartment (Ferreira et al., 1988; Koch et al., 2004; Masoumi et al., 2008). Furthermore, most of the heme produced is required for plastid function in photosynthetic algae, so it makes no sense to localize the final step of heme biosynthesis outside the plastid. The cytosolic localization of FeCH indicates a possible non-canonical function of FeCH, which is not involved in heme synthesis. Previous studies have shown that FeCH can demetallize iron from porphyrin (Taketani et al., 2007). Thus, FeCH can potentially be used for the replacement of iron by other metal ions and acts as an enzyme responsible for the chelation of zinc and cobalt (Dailey et al., 2000).

### 4.3 Comparison of the expression of *UROD* and *CPOX* pseudoparalogs in different environments

The pseudoparalogs of *UROD* and *CPOX* are differentially expressed under nitrate starvation, in knockout lines for aureochrome blue-light receptors, at specific time points in the cell cycle, and in response to high light and high CO_2_ levels. Under these conditions, we can observe two possible correlations in regulatory factors. First, UROD1 is upregulated under nitrate starvation (knockout of nitrate reductase) (McCarthy et al., 2017) after 36 hours and 66 hours of nitrate limitation, and continues till 162 hours. In contrast, *UROD2* and *UROD3* are mostly downregulated within this timeframe (**Supplementary Figure S8**). In contrast, expression of the *CPOX* (pseudo)paralogs is inconsistent across the two experimental conditions (**Supplementary Figure S9**) and therefore inconclusive. It is therefore likely that *UROD* pseudoparalogs are differentially regulated in response to long-term nitrate starvation compared to the other pseudoparalogs.

Second, it has previously been shown that aureochrome 1a, an ochrophyte-wide blue light receptor with a conserved bZIP/LOV domain structure (Takahashi et al., 2007), is differentially expressed across the G1/S cell cycle. We found that the expression patterns of *CPOX* pseudoparalogs were inverse between the 8-hour treatment in the dark (in the G1/S transition phase) and the knock-out mutant of aureochrome1a (**Supplementary Figure S6A**). It has further been shown that the aureochrome1a of *P. tricornutum* can rapidly activate diatom-specific cyclin 2 (dsCYC2) at the G1/S cell cycle transition (Huysman et al., 2013). This induction is consistent with the dark treatments performed by (Matthijs et al., 2017) as cells are exposed to light for a few minutes during their harvesting, and the authors also found that *dsCYC2* had been activated in this treatment. In the case of all *URODs*, the expression at both 0 min and 10 min time points are similar and therefore we did not consider the involvement of aureochrome 1a-dsCyc signaling (**Supplementary Figure S10A**). *UROD3* started to be upregulated at 60 mins, likely due to a different blue light activated signaling pathway. In the case of *CPOX1*, this possibility exists but we only saw a slight increase in the 10 min blue light compared to 0 min in 1aK8 mutant (**Supplementary Figure S10A**). Since *CPOX2* was significantly downregulated in both mutants after 10 min of blue light, which is similar to the *dsCYC2* responding rapidly to re-illumination (especially to blue light for *dsCYC2*), we hypothesize that *CPOX2* could be regulated by blue light via an aureochrome1a-(*dsCYC2)*-dependent signaling pathway (**Supplementary Figure S10B**).

Finally, in an experiment comparing high light (940 µmol photons/m^2^/s) and low light (20 µmol photons/m2/s) (Agarwal et al., 2021), CPOX1 was drastically downregulated and *CPOX2* slightly downregulated, while *CPOX3* was upregulated in the high light conditions (**Supplementary Figure S6A**). This indicates a significant role of CPOX3 in response to high-light stress. The treatments (BioProject Accession: PRJEB34512) were maintained in a semi-continuous cultivation device for eight months, *CPOX1* is therefore likely to adapt better to prolonged exposure to a high CO_2_ environment than *CPOX2* and *CPOX3*.

The *URODs* and *CPOXs* genes are differentially expressed under the above-mentioned condition. In most cases, these pseudoparalogs are expressed together and are colocalized in the plastids. This suggests that the enzyme encoded by different pseudoparalogs could hypothetically form autonomous dimers with others. We test this possibility using AlphaFold 2.0 and the putative homo- and heterodimers can be predicted *in silico* (**Supplementary Figure S11**). However, whether these evolutionarily diverse enzymes could indeed work together, remains to be explored in future studies.

### 4.4 Evolution of heme biosynthesis in the complex plastids of diatom *P. tricornutum*

**Figure 6:**
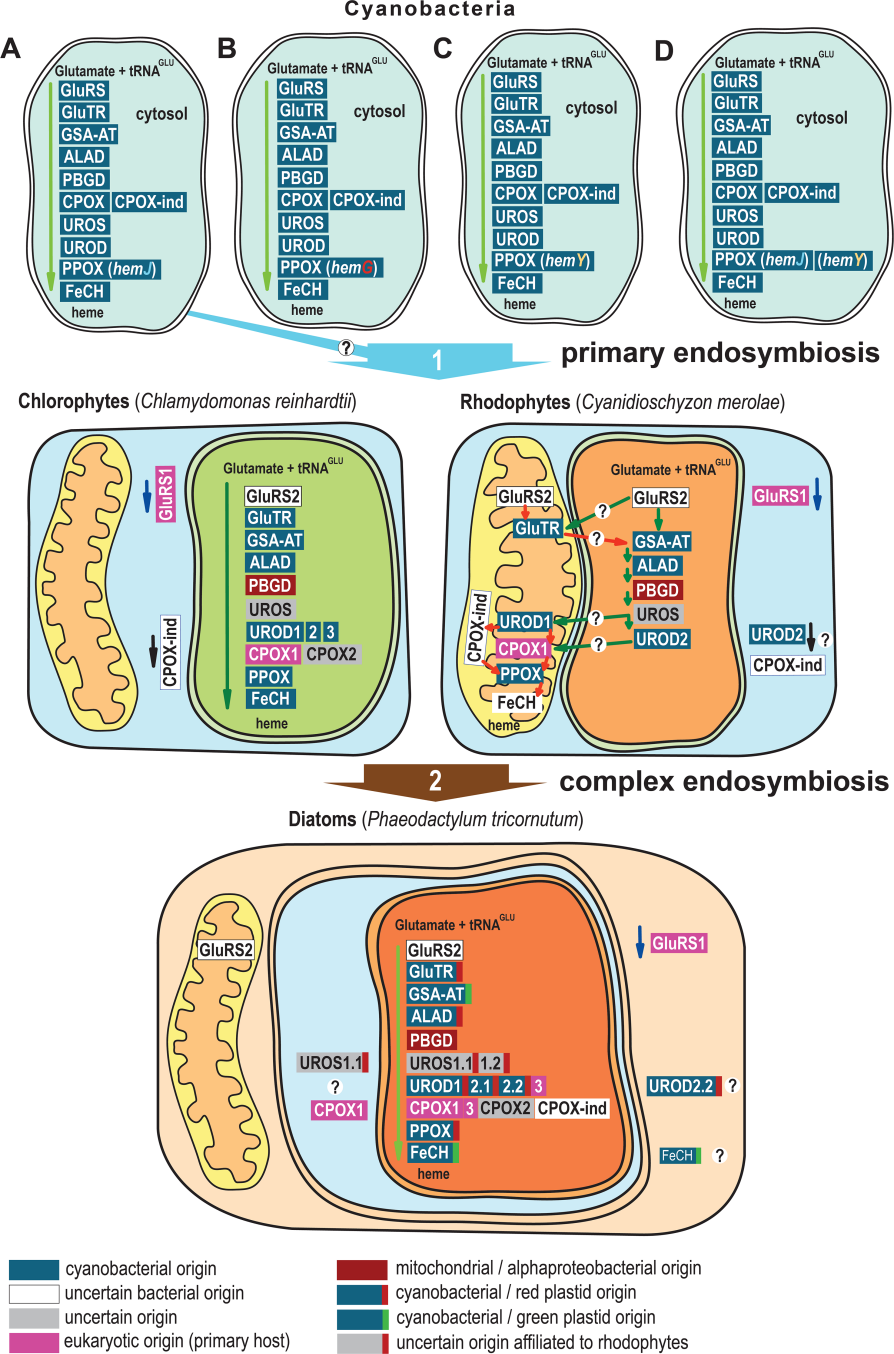
Evolution and localization of heme biosynthesis in the complex plastid of diatom *P. tricornutum*. **(A)** Most cyanobacteria use PPOX (*hemJ*) including the closest existing relatives of plastids, *Gloeomargarita lithophora*. **(B)** Prochlorococcus marinus strains MIT9215 and MIT9515 use the oxygen-independent PPOX (*hemG*) **(C)** *Synechococcus JA-2-3B’a(2–13), Synechococcus* sp. *JA-3-3Ab, Trichodesmium erythraeum* and *Thermosynechococcus elongatus* habor *HemY* **(D)** *Synechococcus elongatus, Synechocystis* sp. *PCC 6308, Synechococcus* sp. *PCC 7002, Microcystis_aeruginosa, Cyanothece_PCC, Gloeobacter violaceus, Nostoc* sp. *PCC 7120, Nostoc punctiforme PCC 73102*, and *Trichormus variabilis ATCC 29413* possess both *hemJ* and *hemY* for PPOX. Primary algae acquired genes coding GluTR, GSA-AT, ALAD, UROD, PPOX, and FeCH (only chlorophytes) from cyanobacteria. Since the eukaryotic PPOX is encoded by *hemY*, the cyanobacterial ancestor of plastids might have had PPOX (*hemY*) but that it is likely lost in most modern cyanobacterial descendants. Diatoms obtained many heme pathway genes via complex endosymbiotic, or in other horizontal gene transfers. The *P. tricornutum GluTR*, *ALAD*, *UROS1*, *UROD1*, *UROD2* and *PPOX* are of red algal origin, and *GSA-AT*, *UROD2*, and *FeCH* are of green algal origin, the heme biosynthesis pathway in diatoms is therefore an evolutionary mosaic (Cihlář et al., 2016). The heme pathway in cyanobacteria is summarized from (Kobayashi et al., 2014). The localization of C*hlamydomonas reinhardtii* heme pathway is based on (van Lis et al., 2005; Wang et al., 2023). Heme pathway enzyme localizations in *Cyanidioschyzon merolae* are based on (Watanabe et al., 2013), and were checked with TargetP 2.0 (Almagro Armenteros et al., 2019a). The depicted localizations of heme biosynthesis enzymes in the diatom *P. tricornutum* summarize the results of this study. The phylogenetic classification of the genes is based on (Kořený and Oborník, 2011; Cihlář et al., 2016; Sharaf et al., 2019) and EnsemblPlants gene tree (Bolser et al., 2017).

*P. tricornutum* obtained many heme pathway genes via a complex endosymbiotic gene transfer. Most of the genes could be traced back to cyanobacterial ancestry, and others are of alphaproteobacterial (*PBGD*) or eukaryotic origin (*GluRS1, UROD3, CPOX1* and *CPOX3*) (**Figure 6**). Diatoms habor a rhodophyte-derived complex plastid. Many genes that are originally cyanobacterial, were acquired by diatoms indirectly, via the eukaryotic ancestor of the complex plastid, which had already acquired the genes from the cyanobacterial ancestor of all plastids. These genes (*GluTR*, *ALAD*, *UROD1*, *UROD2* and *PPOX*) show corresponding phylogenies. Other genes, however, (*GSA-AT*, *UROD3*, and *FeCH*), based on molecular phylogenies, must be of independent origin. While they also go back to cyanobacteria, gene trees suggest they were acquired from green algae, implying a more complicated scenario of diatom plastid origin than “simple” endosymbiotic plastid acquisition from a red alga. Diatoms, primary algae and other eukaryotic PPOXs are encoded by *hemY.* However, the closest existing cyanobacterial relative of plastids, uses PPOXs encoded by *hemJ*. Nevertheless, PPOX (*hemY)* can still be found in other groups of cyanobacteria (**Figures 6C, D**). The cyanobacterial ancestor of plastids might have had PPOX (*hemY*), which however likely was lost in most extant groups of cyanobacteria.

## 5 Conclusion

The enzymes responsible for the biosynthesis of heme in the diatom *P. tricornutum* have been shown to be an evolutionary mosaic reflecting past endosymbiotic events (**Figure 6**). The entire metabolic pathway is mainly located in the plastid (**Figure 6**), but not all enzymes appear to be exclusively localized in the plastid; UROS1.1 and CPOX1 show a possible dual localization in the plastid and the PPC. Moreover, UROD2.2 and FeCH may be localized in both the plastid and the cytosol. Several pseudoparalogs of the UROD and CPOX genes show no spatial differences in their localization but are transcriptionally activated under different environmental conditions. Analysis of RNA-seq data suggests that the *UROD* paralogs respond differently to nitrate starvation, and the *CPOX* paralogs show differential expression patterns between cell cycle, high light, high CO_2_ and blue light. Their similar protein structures, co-expression in the majority of conditions, and co-localization in the same cellular compartments suggest that they retain at least partially redundant biochemical roles and that homo- or heterodimers can hypothetically be formed in the plastids. However, given the differences in expression of the investigated pseudoparalogs, it also seems clear that these genes are not entirely redundant but instead underwent some regulatory differentiation.

## Data Availability

The datasets presented in this study can be found in online repositories. The names of the repository/repositories and accession number(s) can be found in the article/**Supplementary Material**.
