# Supplementary material for: Localization of heme biosynthesis in the diatom Phaeodactylum tricornutum and differential expression of multi-copy enzymes
Source: Front Plant Sci. 2025 Mar 4;16:1537037. doi: 10.3389/fpls.2025.1537037 (PMC11914136; doi:10.3389/fpls.2025.1537037)
Supplement: Supplementary file 3 [file Presentation1.pptx]

## Slide 1
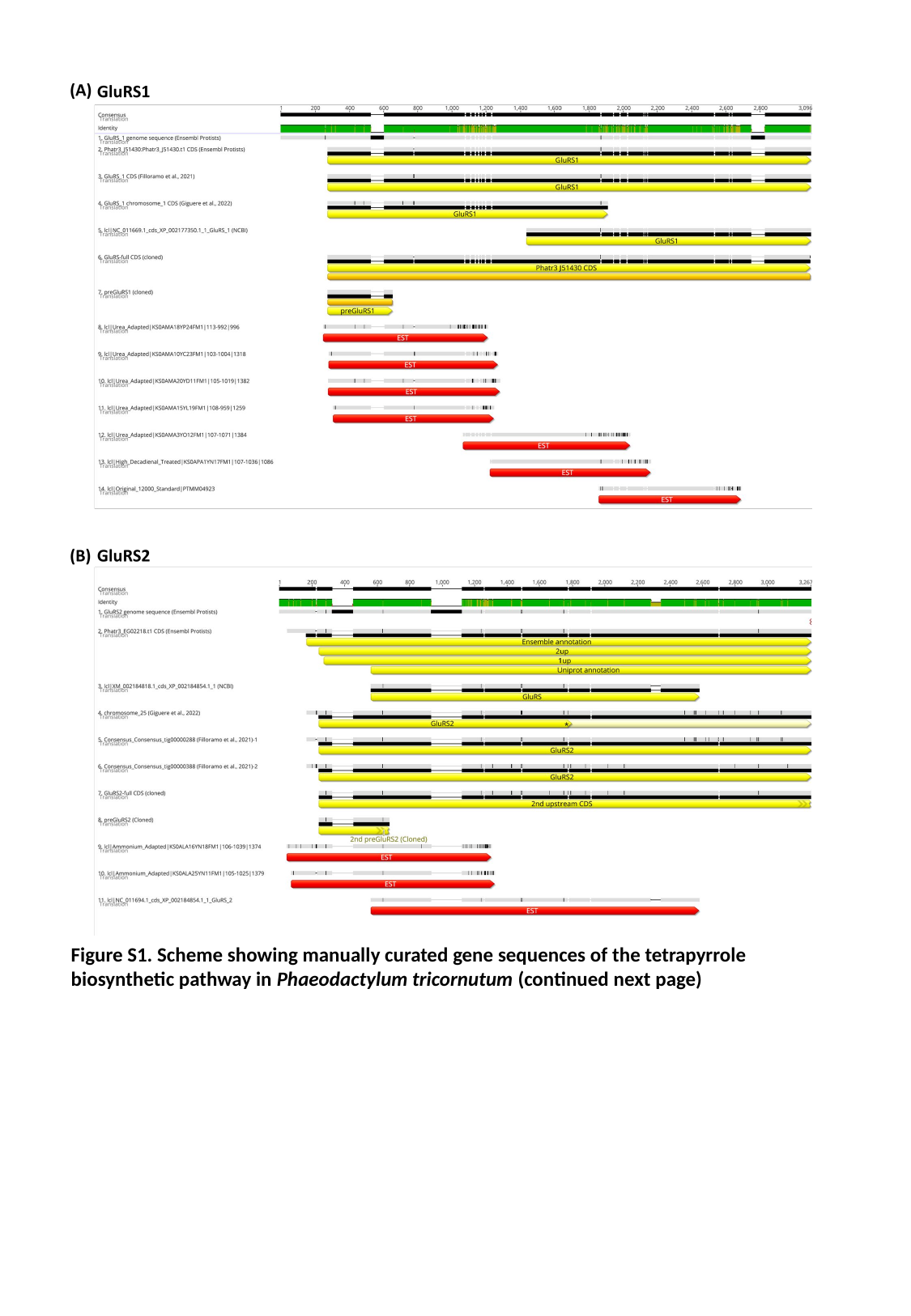

Figure S1. Scheme showing manually curated gene sequences of the tetrapyrrole biosynthetic pathway in Phaeodactylum tricornutum (continued next page)

## Slide 2
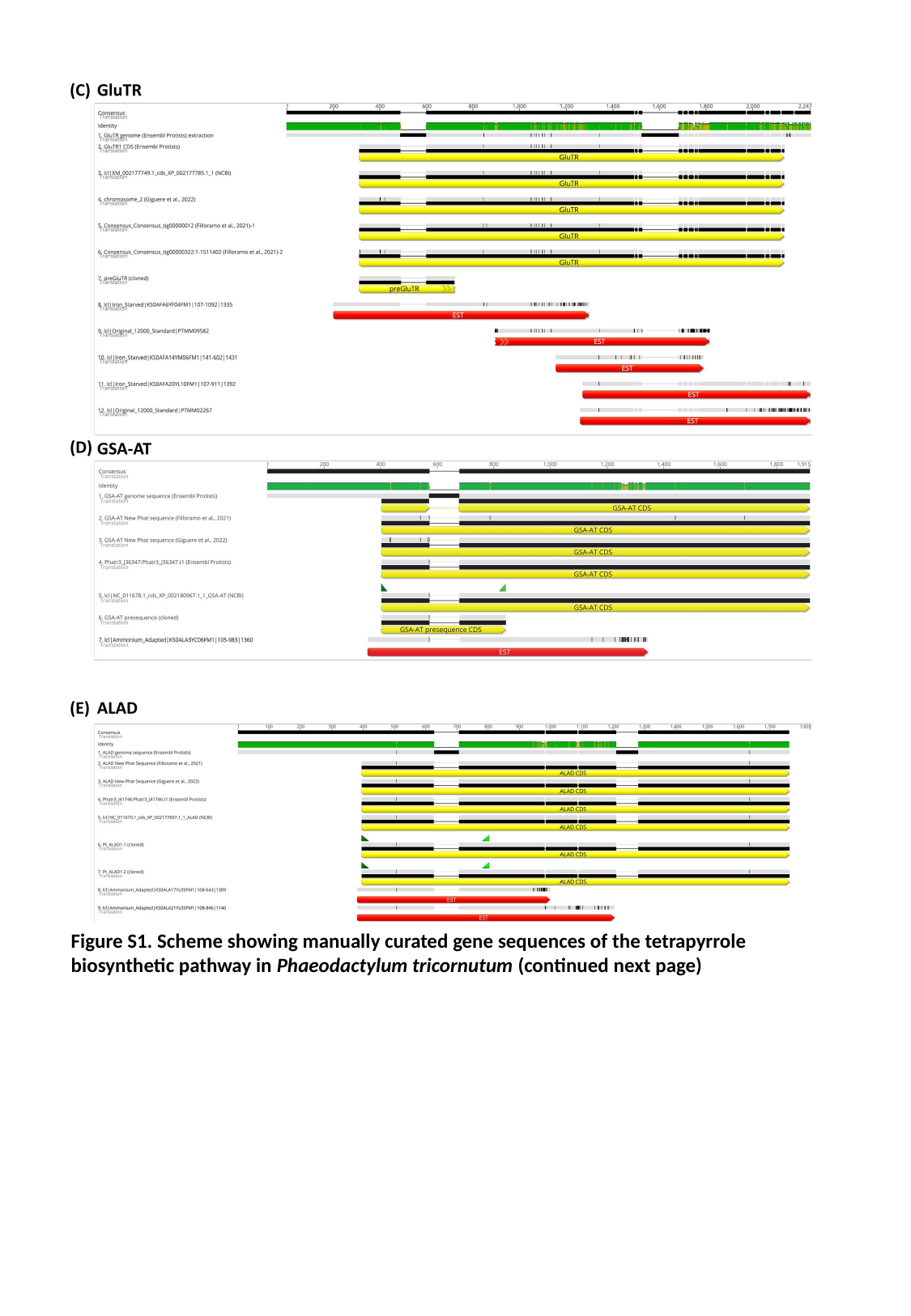

Figure S1. Scheme showing manually curated gene sequences of the tetrapyrrole biosynthetic pathway in Phaeodactylum tricornutum (continued next page)

## Slide 3
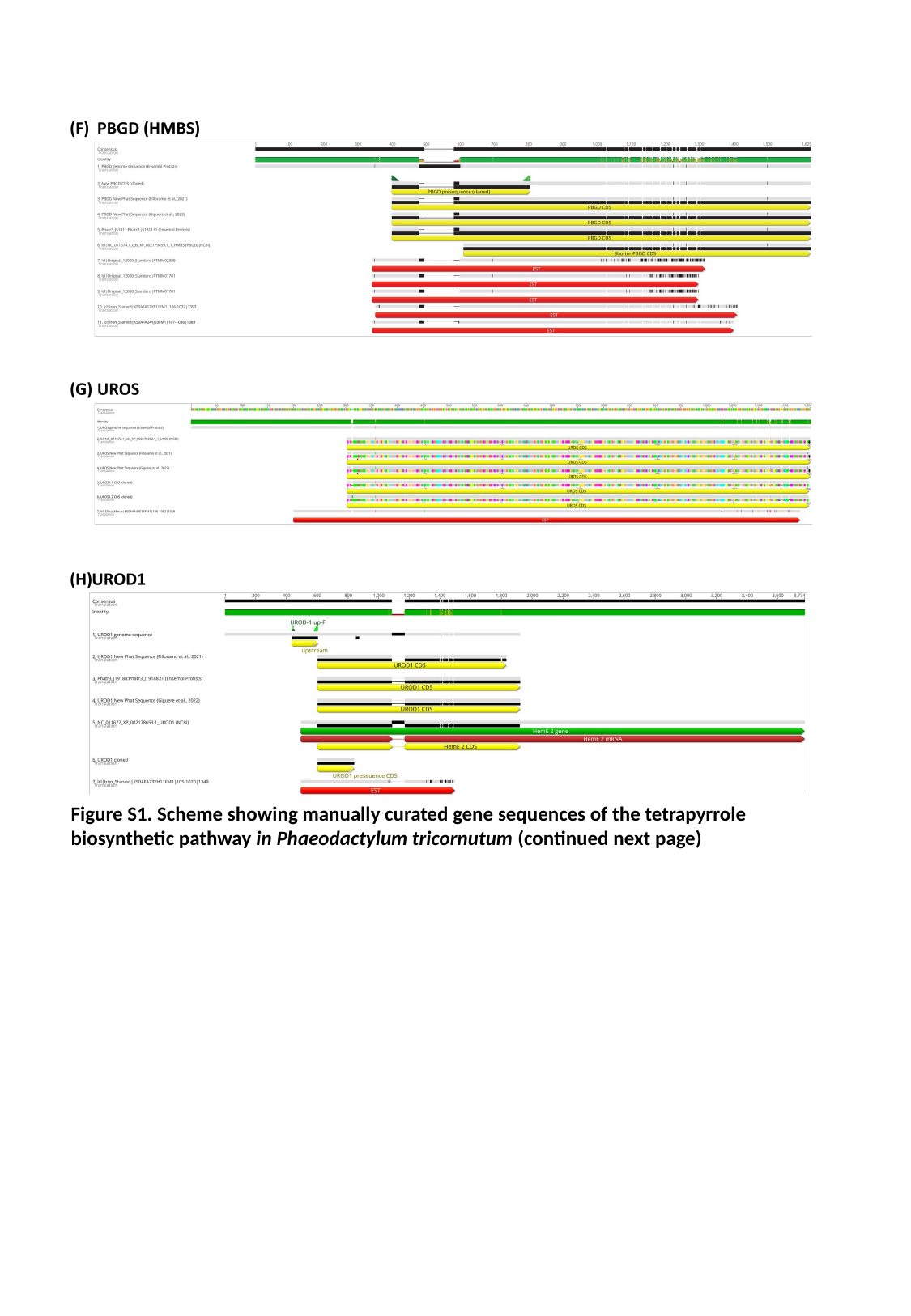

Figure S1. Scheme showing manually curated gene sequences of the tetrapyrrole biosynthetic pathway in Phaeodactylum tricornutum (continued next page)

## Slide 4
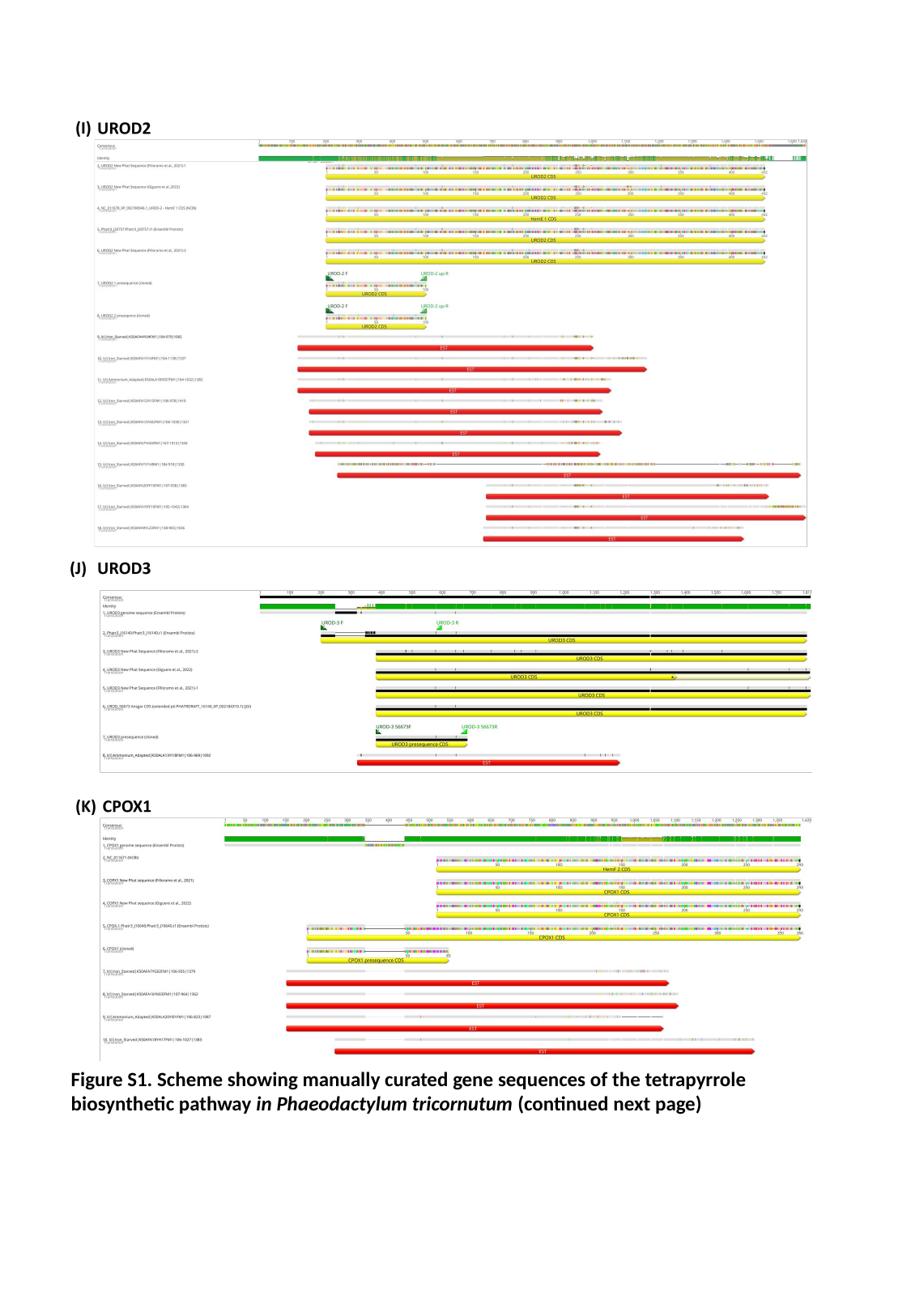

Figure S1. Scheme showing manually curated gene sequences of the tetrapyrrole biosynthetic pathway in Phaeodactylum tricornutum (continued next page)

## Slide 5
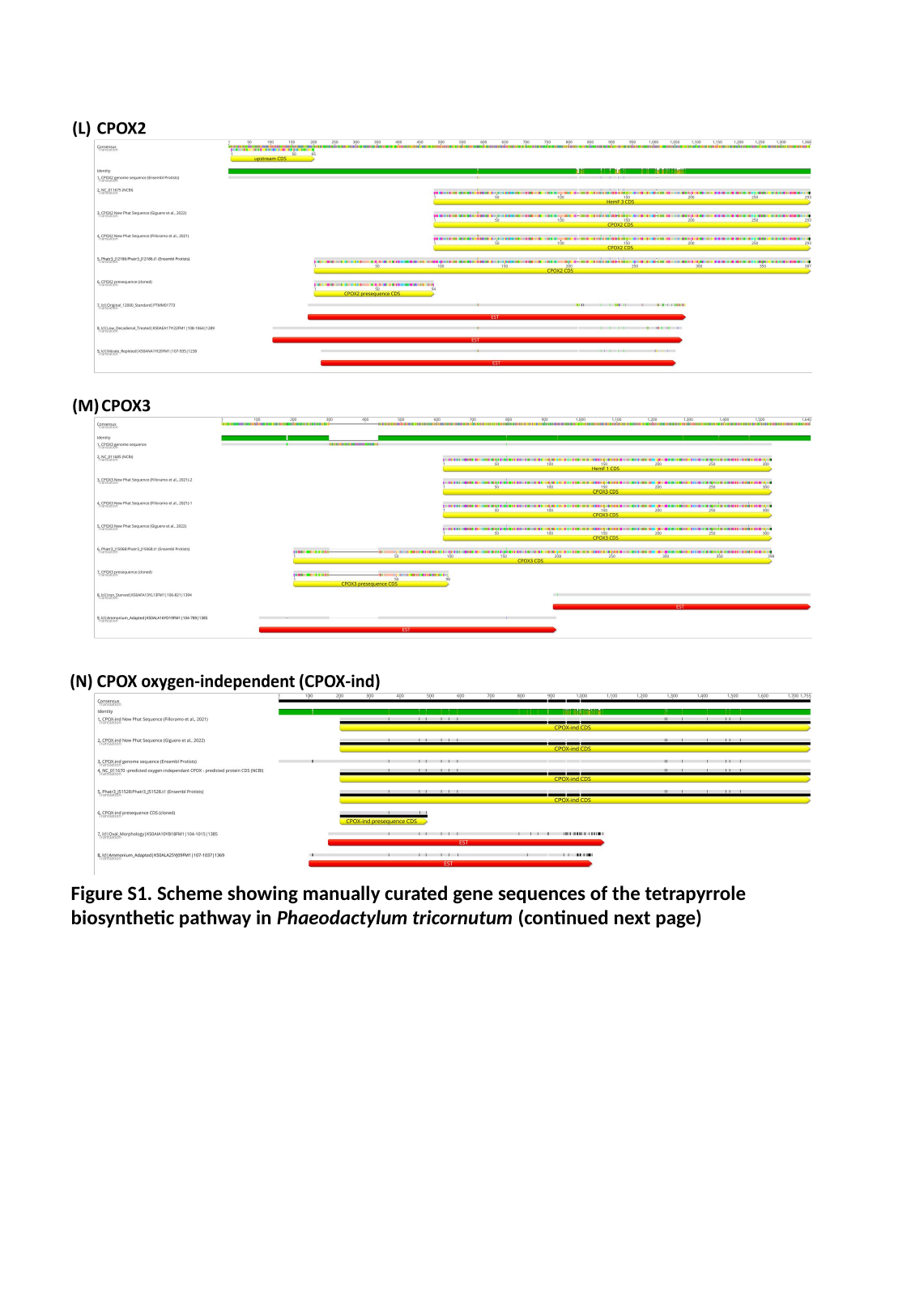

Figure S1. Scheme showing manually curated gene sequences of the tetrapyrrole biosynthetic pathway in Phaeodactylum tricornutum (continued next page)

## Slide 6
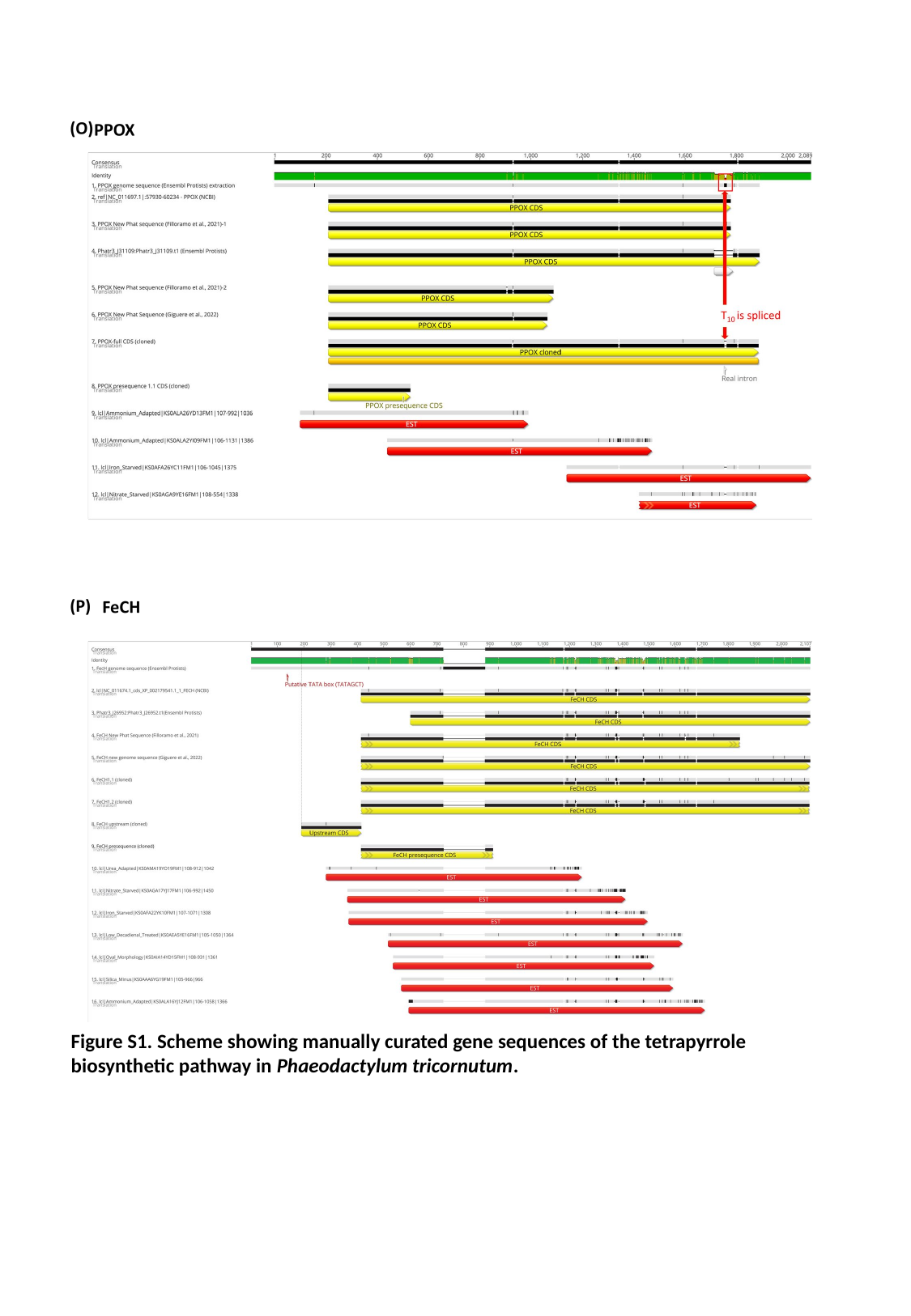

Figure S1. Scheme showing manually curated gene sequences of the tetrapyrrole biosynthetic pathway in Phaeodactylum tricornutum.

## Slide 7
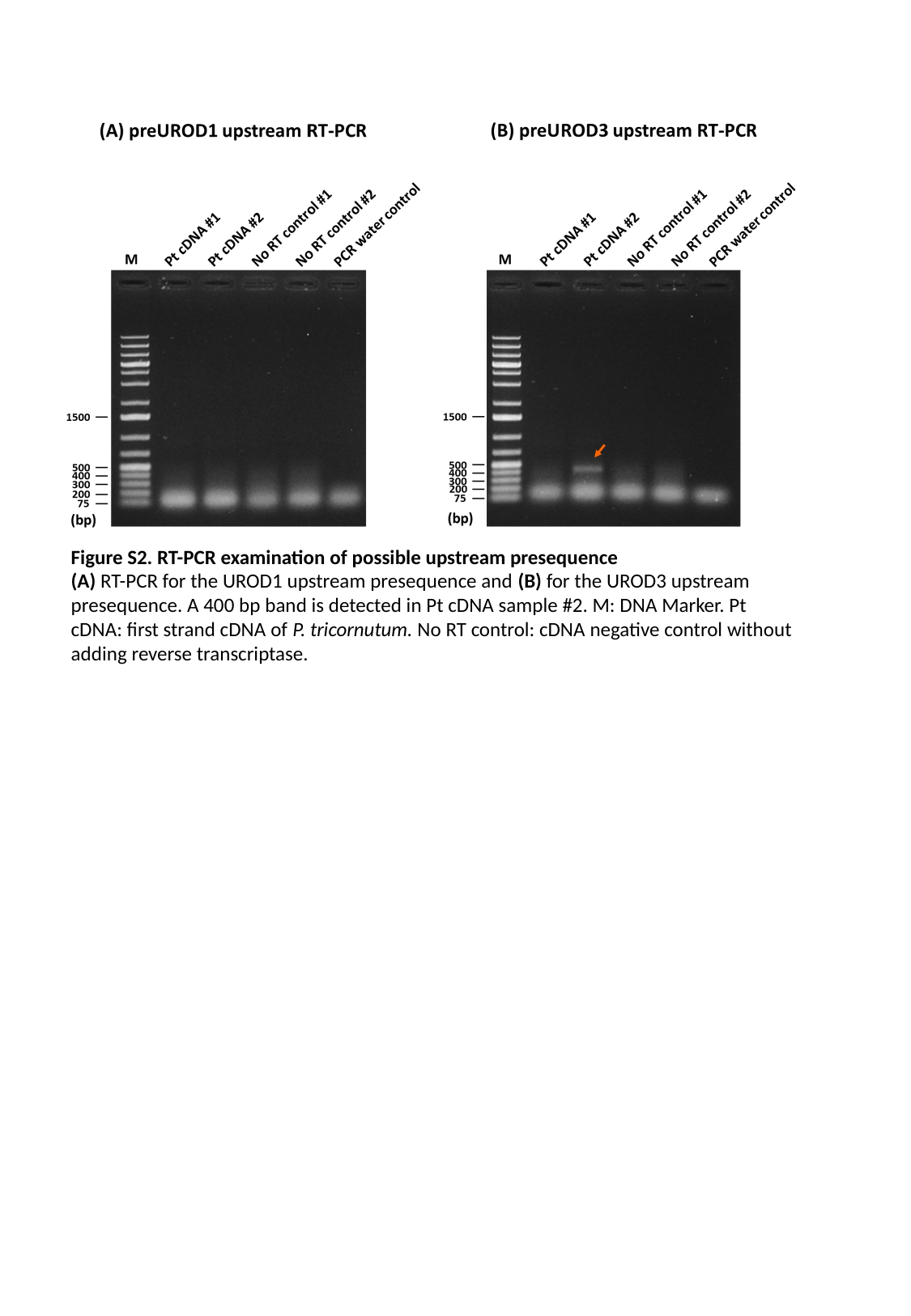

Figure S2. RT-PCR examination of possible upstream presequence
(A) RT-PCR for the UROD1 upstream presequence and (B) for the UROD3 upstream presequence. A 400 bp band is detected in Pt cDNA sample #2. M: DNA Marker. Pt cDNA: first strand cDNA of P. tricornutum. No RT control: cDNA negative control without adding reverse transcriptase.

## Slide 8
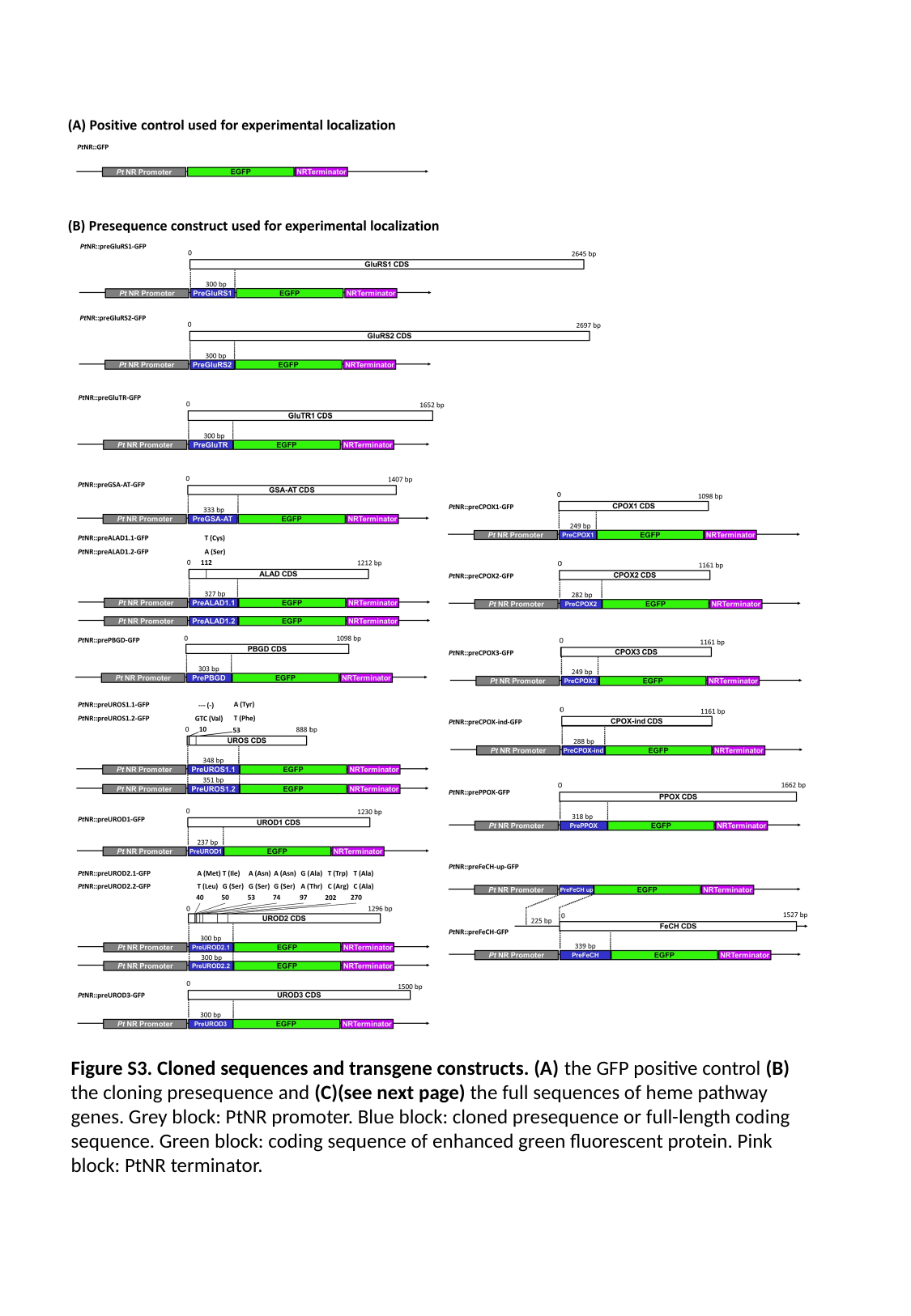

Figure S3. Cloned sequences and transgene constructs. (A) the GFP positive control (B) the cloning presequence and (C)(see next page) the full sequences of heme pathway genes. Grey block: PtNR promoter. Blue block: cloned presequence or full-length coding sequence. Green block: coding sequence of enhanced green fluorescent protein. Pink block: PtNR terminator.

## Slide 9
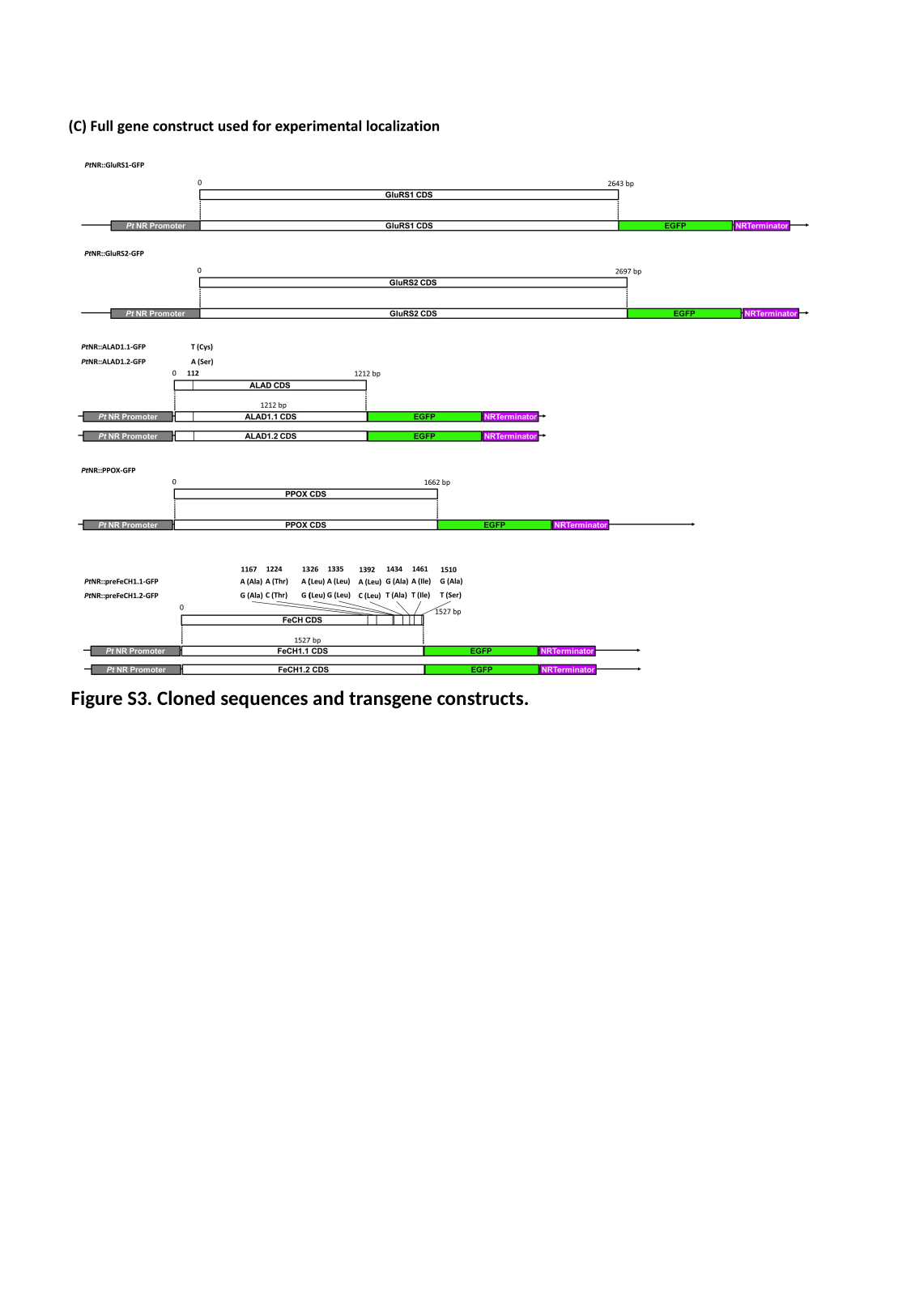

Figure S3. Cloned sequences and transgene constructs.
