# Supplementary material for: Localization of heme biosynthesis in the diatom Phaeodactylum tricornutum and differential expression of multi-copy enzymes
Source: Front Plant Sci. 2025 Mar 4;16:1537037. doi: 10.3389/fpls.2025.1537037 (PMC11914136; doi:10.3389/fpls.2025.1537037)
Supplement: Supplementary file 4 [file Presentation2.pptx]

## Slide 1
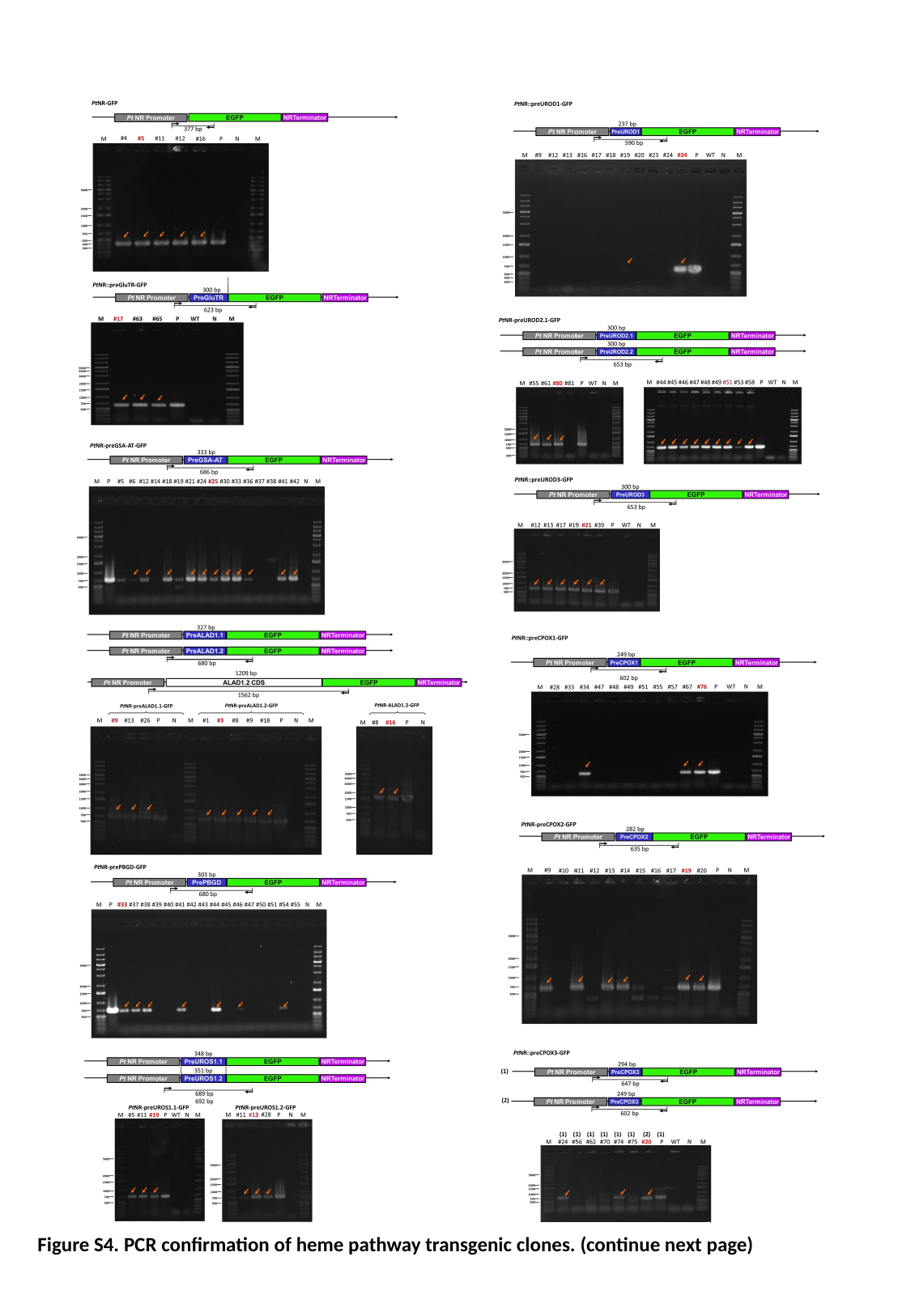

Figure S4. PCR confirmation of heme pathway transgenic clones. (continue next page)

## Slide 2
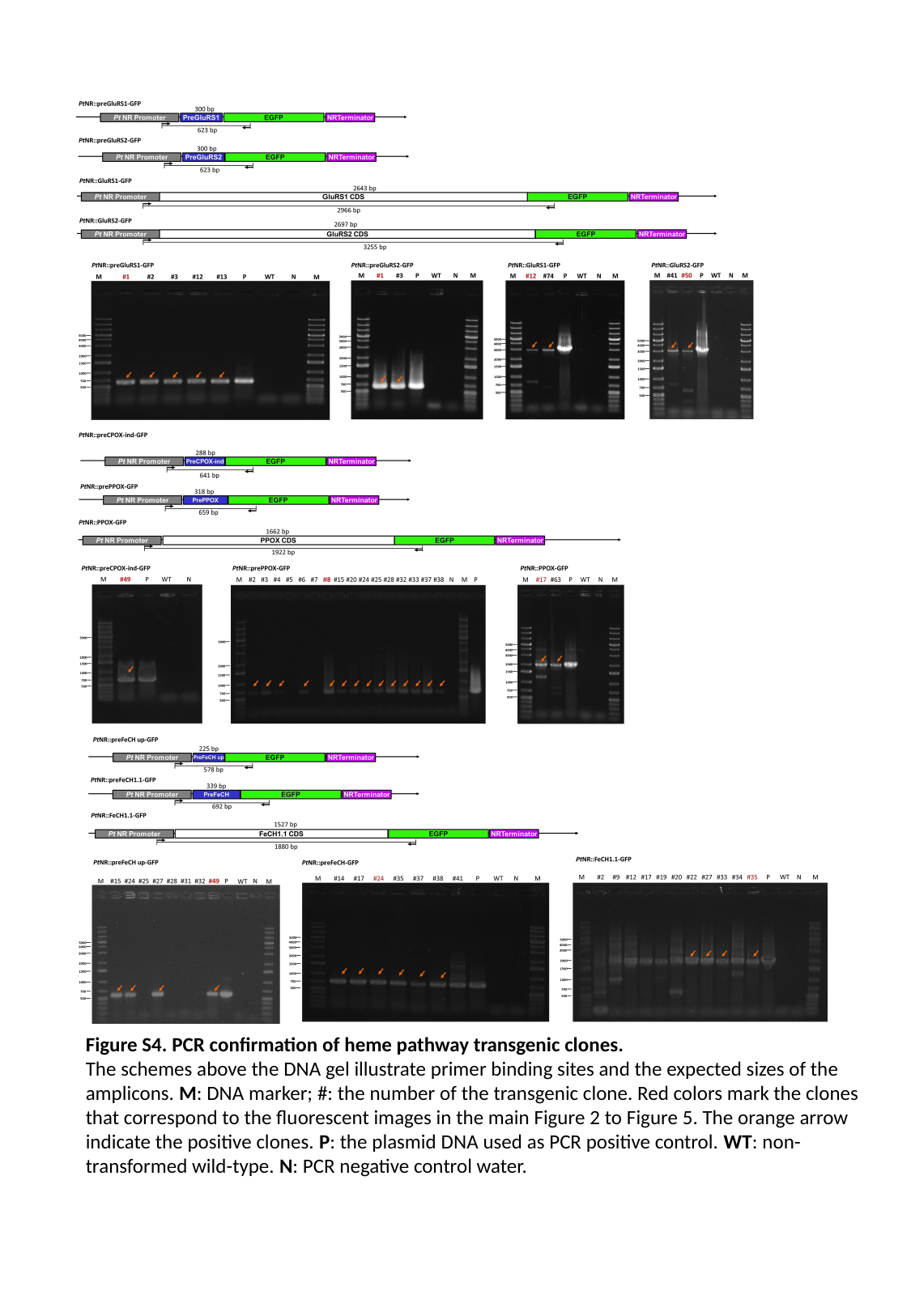

Figure S4. PCR confirmation of heme pathway transgenic clones.
The schemes above the DNA gel illustrate primer binding sites and the expected sizes of the amplicons. M: DNA marker; #: the number of the transgenic clone. Red colors mark the clones that correspond to the fluorescent images in the main Figure 2 to Figure 5. The orange arrow indicate the positive clones. P: the plasmid DNA used as PCR positive control. WT: non-transformed wild-type. N: PCR negative control water.

## Slide 3
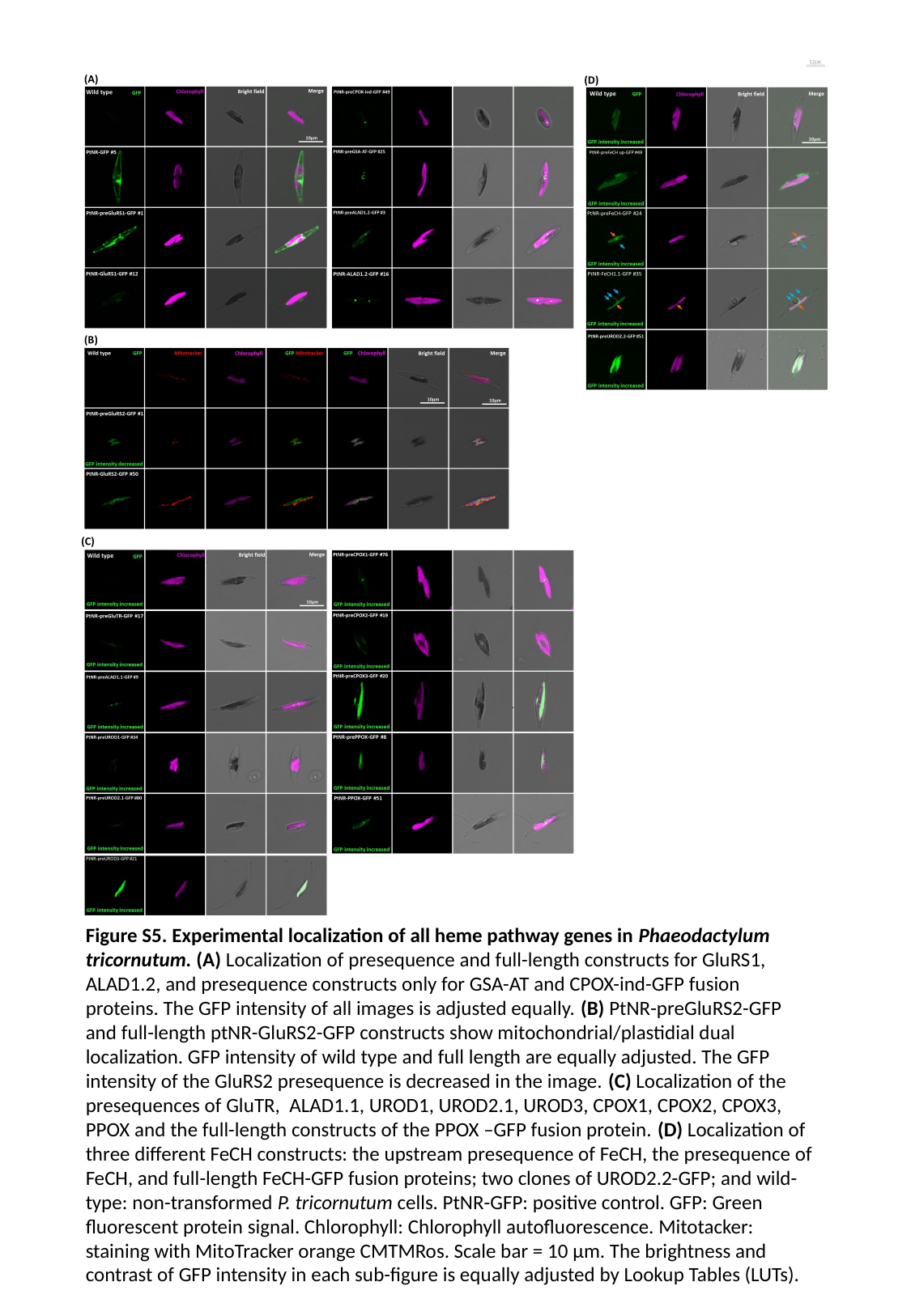

Figure S5. Experimental localization of all heme pathway genes in Phaeodactylum tricornutum. (A) Localization of presequence and full-length constructs for GluRS1, ALAD1.2, and presequence constructs only for GSA-AT and CPOX-ind-GFP fusion proteins. The GFP intensity of all images is adjusted equally. (B) PtNR-preGluRS2-GFP and full-length ptNR-GluRS2-GFP constructs show mitochondrial/plastidial dual localization. GFP intensity of wild type and full length are equally adjusted. The GFP intensity of the GluRS2 presequence is decreased in the image. (C) Localization of the presequences of GluTR, ALAD1.1, UROD1, UROD2.1, UROD3, CPOX1, CPOX2, CPOX3, PPOX and the full-length constructs of the PPOX –GFP fusion protein. (D) Localization of three different FeCH constructs: the upstream presequence of FeCH, the presequence of FeCH, and full-length FeCH-GFP fusion proteins; two clones of UROD2.2-GFP; and wild-type: non-transformed P. tricornutum cells. PtNR-GFP: positive control. GFP: Green fluorescent protein signal. Chlorophyll: Chlorophyll autofluorescence. Mitotacker: staining with MitoTracker orange CMTMRos. Scale bar = 10 µm. The brightness and contrast of GFP intensity in each sub-figure is equally adjusted by Lookup Tables (LUTs).

## Slide 4
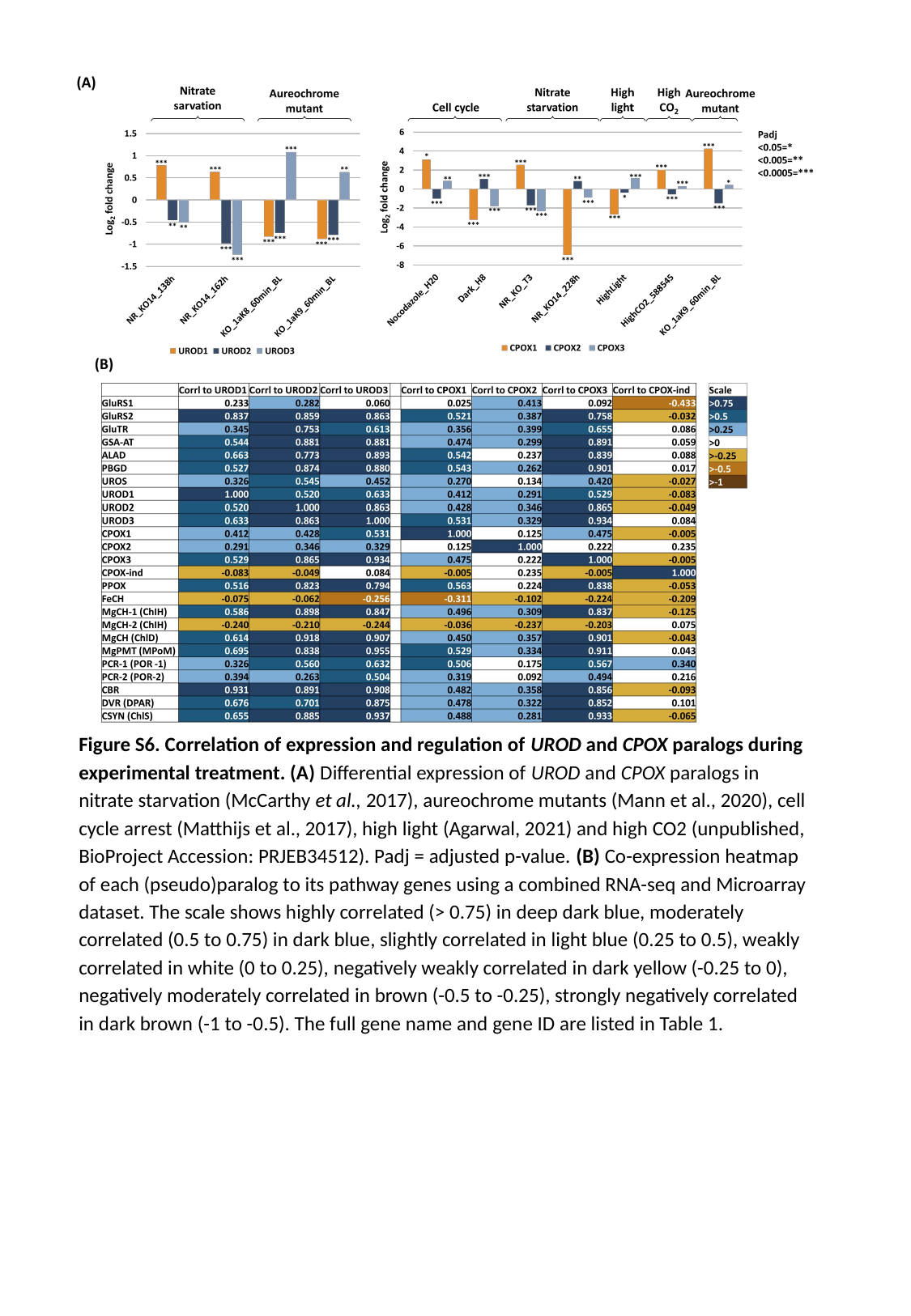

Figure S6. Correlation of expression and regulation of UROD and CPOX paralogs during experimental treatment. (A) Differential expression of UROD and CPOX paralogs in nitrate starvation (McCarthy et al., 2017), aureochrome mutants (Mann et al., 2020), cell cycle arrest (Matthijs et al., 2017), high light (Agarwal, 2021) and high CO2 (unpublished, BioProject Accession: PRJEB34512). Padj = adjusted p-value. (B) Co-expression heatmap of each (pseudo)paralog to its pathway genes using a combined RNA-seq and Microarray dataset. The scale shows highly correlated (> 0.75) in deep dark blue, moderately correlated (0.5 to 0.75) in dark blue, slightly correlated in light blue (0.25 to 0.5), weakly correlated in white (0 to 0.25), negatively weakly correlated in dark yellow (-0.25 to 0), negatively moderately correlated in brown (-0.5 to -0.25), strongly negatively correlated in dark brown (-1 to -0.5). The full gene name and gene ID are listed in Table 1.

## Slide 5
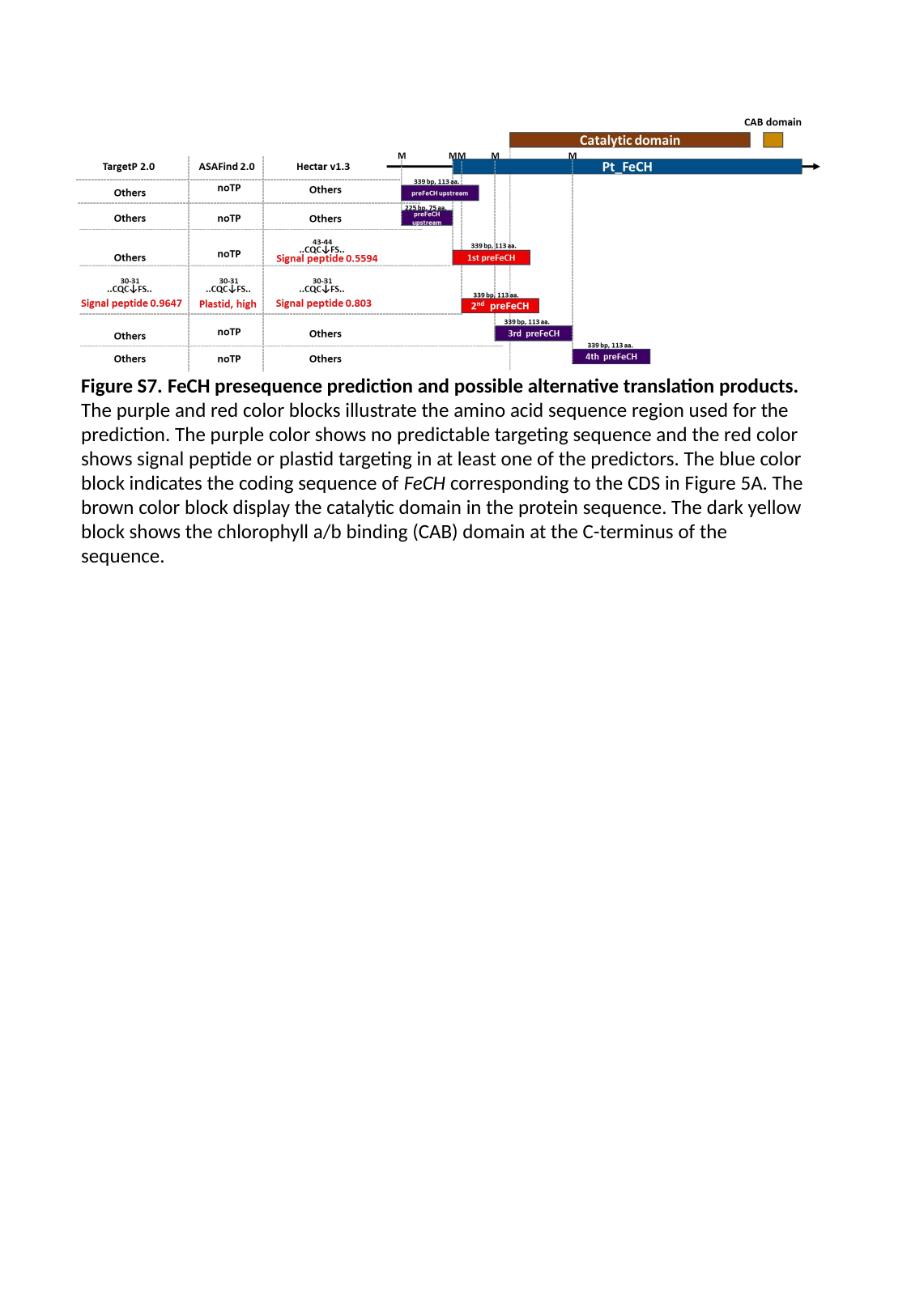

Figure S7. FeCH presequence prediction and possible alternative translation products. The purple and red color blocks illustrate the amino acid sequence region used for the prediction. The purple color shows no predictable targeting sequence and the red color shows signal peptide or plastid targeting in at least one of the predictors. The blue color block indicates the coding sequence of FeCH corresponding to the CDS in Figure 5A. The brown color block display the catalytic domain in the protein sequence. The dark yellow block shows the chlorophyll a/b binding (CAB) domain at the C-terminus of the sequence.

## Slide 6
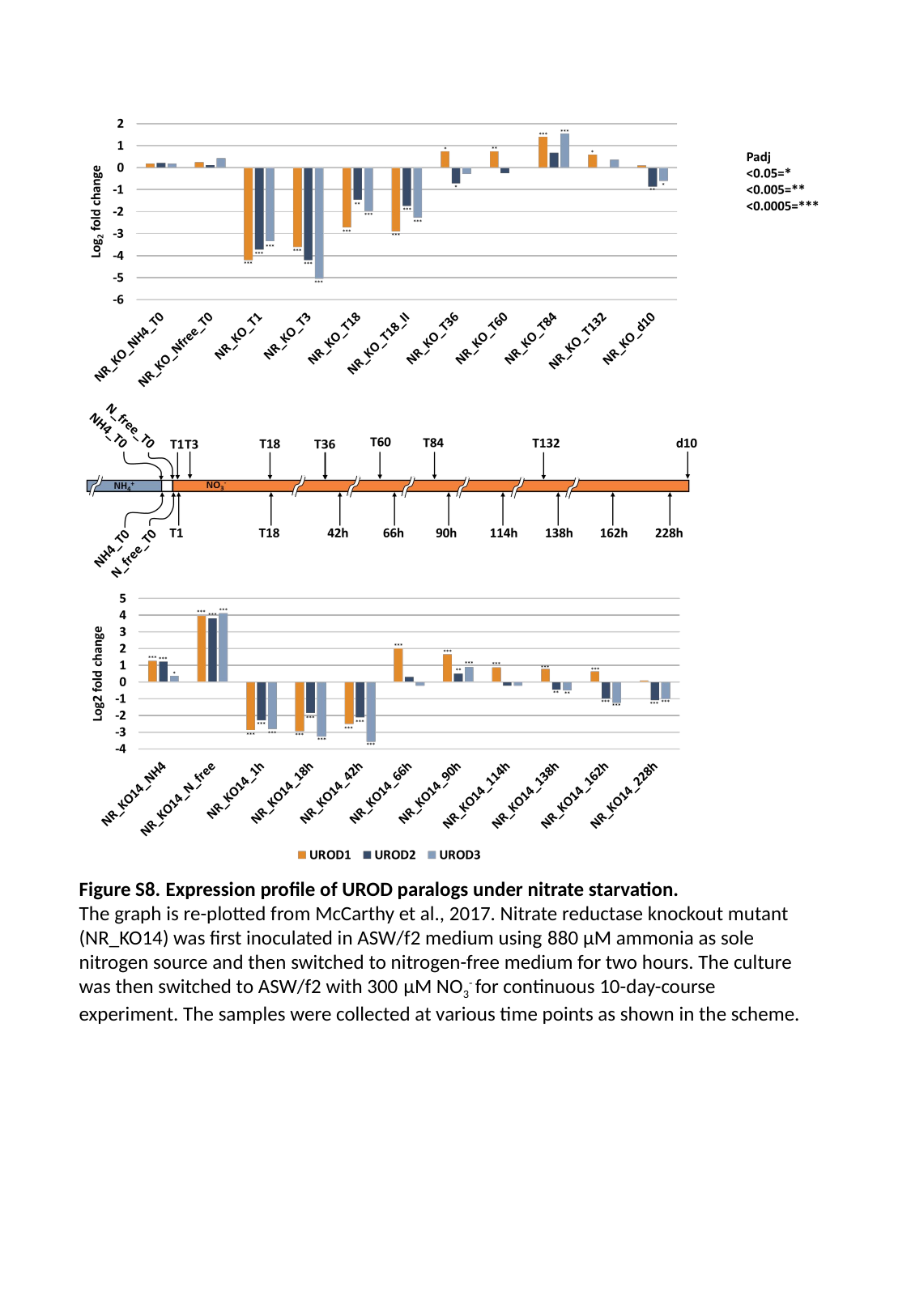

Figure S8. Expression profile of UROD paralogs under nitrate starvation.
The graph is re-plotted from McCarthy et al., 2017. Nitrate reductase knockout mutant (NR_KO14) was first inoculated in ASW/f2 medium using 880 μM ammonia as sole nitrogen source and then switched to nitrogen-free medium for two hours. The culture was then switched to ASW/f2 with 300 μM NO3- for continuous 10-day-course experiment. The samples were collected at various time points as shown in the scheme.

## Slide 7
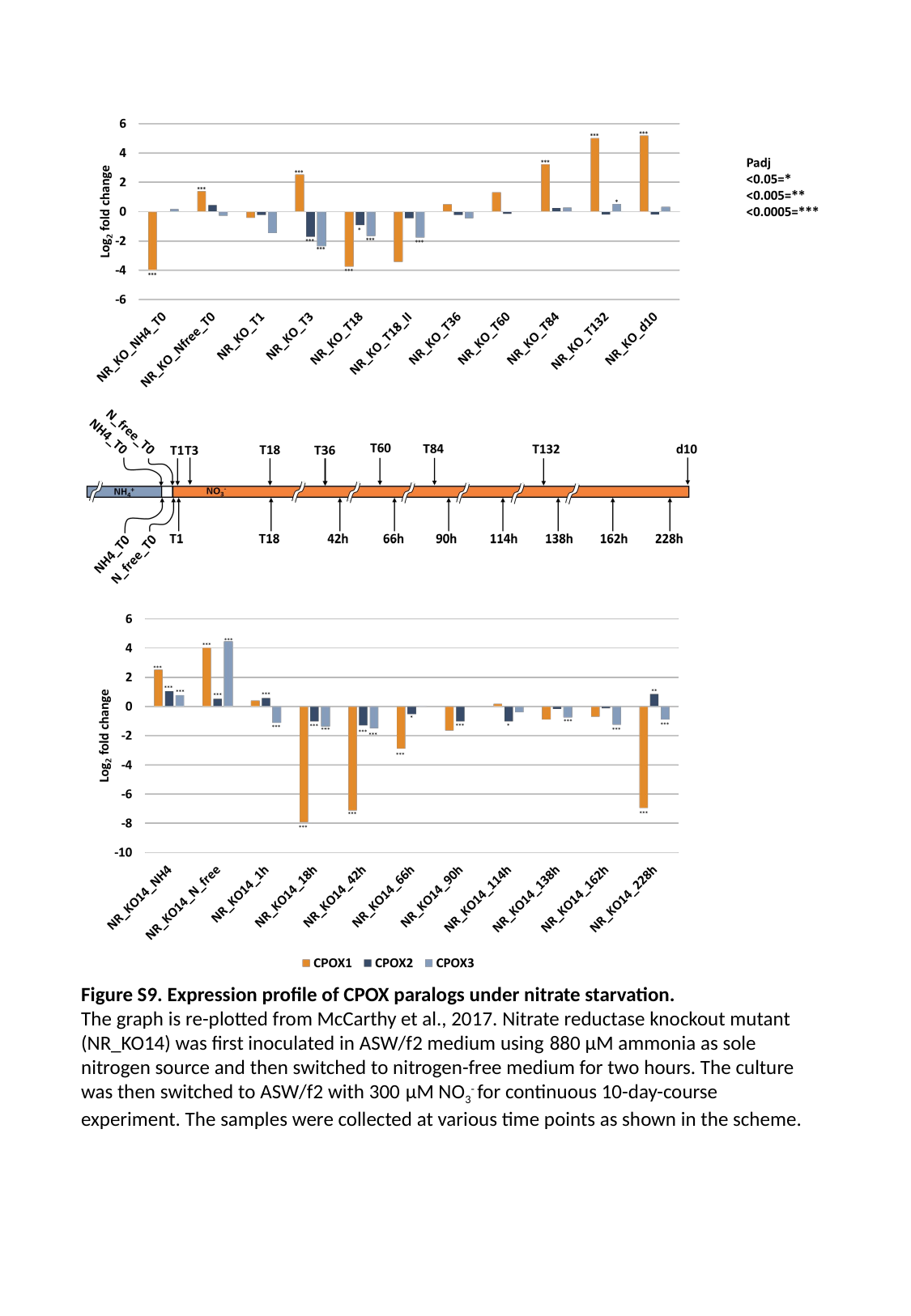

Figure S9. Expression profile of CPOX paralogs under nitrate starvation.
The graph is re-plotted from McCarthy et al., 2017. Nitrate reductase knockout mutant (NR_KO14) was first inoculated in ASW/f2 medium using 880 μM ammonia as sole nitrogen source and then switched to nitrogen-free medium for two hours. The culture was then switched to ASW/f2 with 300 μM NO3- for continuous 10-day-course experiment. The samples were collected at various time points as shown in the scheme.

## Slide 8
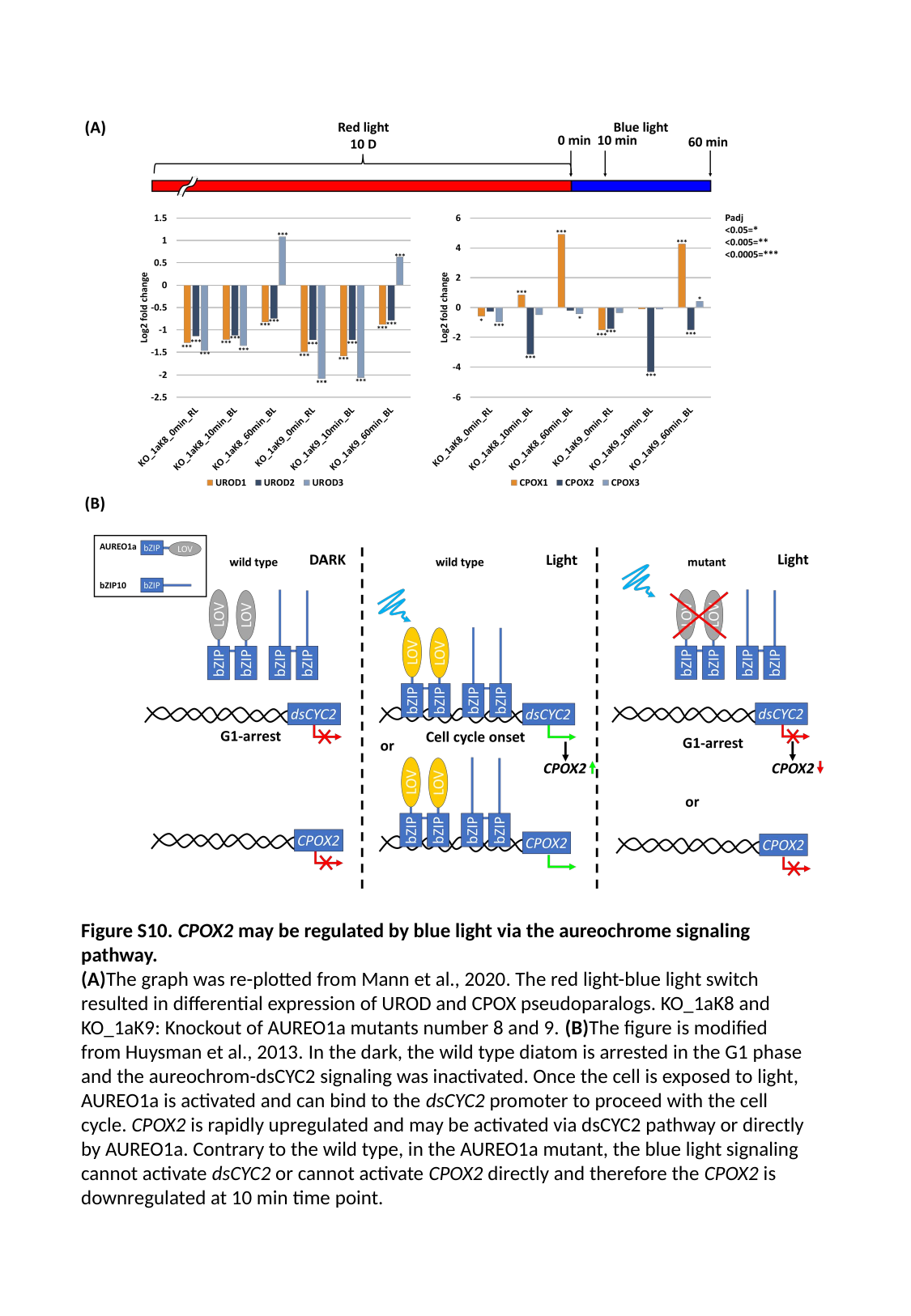

Figure S10. CPOX2 may be regulated by blue light via the aureochrome signaling pathway.
(A)The graph was re-plotted from Mann et al., 2020. The red light-blue light switch resulted in differential expression of UROD and CPOX pseudoparalogs. KO_1aK8 and KO_1aK9: Knockout of AUREO1a mutants number 8 and 9. (B)The figure is modified from Huysman et al., 2013. In the dark, the wild type diatom is arrested in the G1 phase and the aureochrom-dsCYC2 signaling was inactivated. Once the cell is exposed to light, AUREO1a is activated and can bind to the dsCYC2 promoter to proceed with the cell cycle. CPOX2 is rapidly upregulated and may be activated via dsCYC2 pathway or directly by AUREO1a. Contrary to the wild type, in the AUREO1a mutant, the blue light signaling cannot activate dsCYC2 or cannot activate CPOX2 directly and therefore the CPOX2 is downregulated at 10 min time point.

## Slide 9
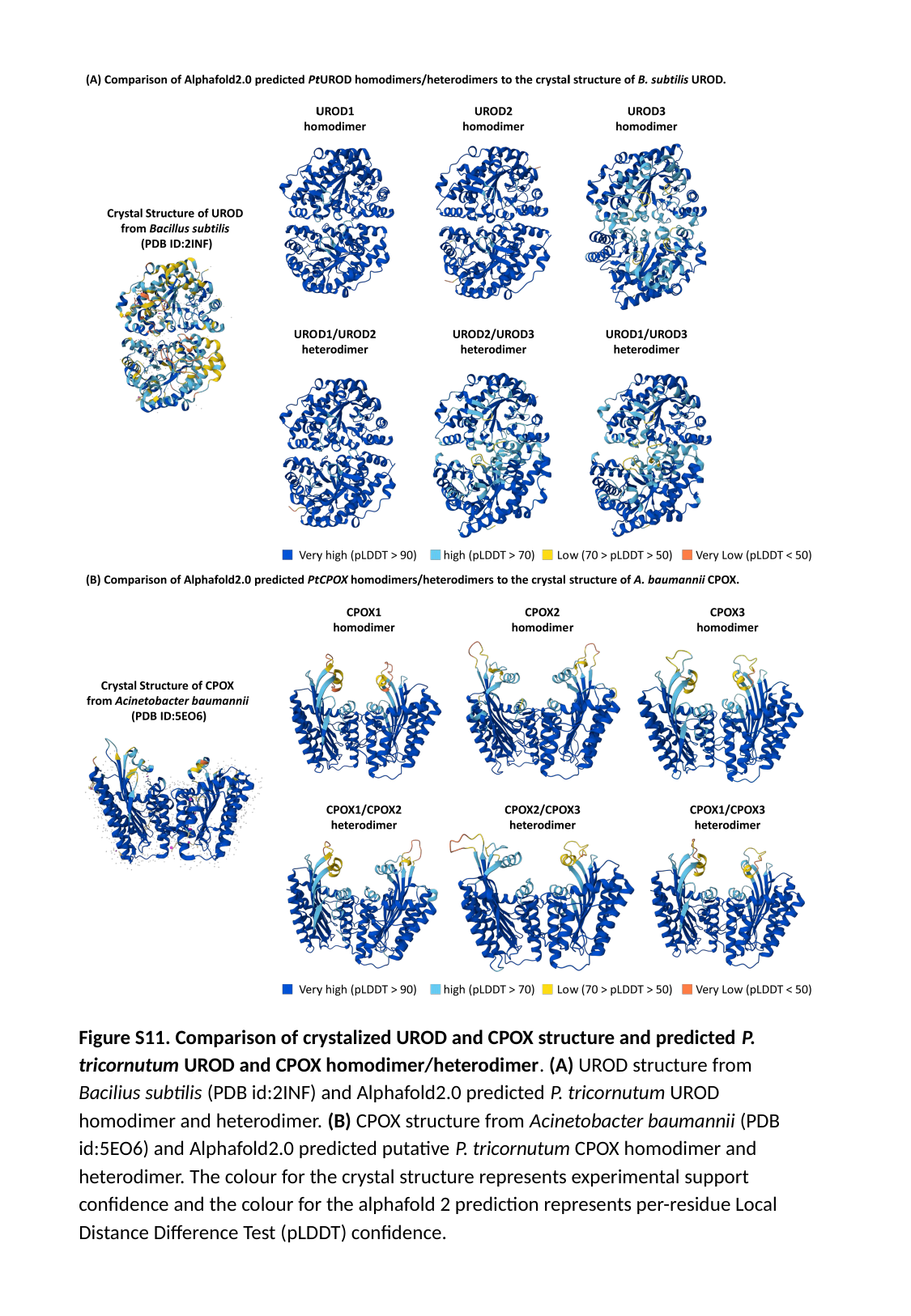

Figure S11. Comparison of crystalized UROD and CPOX structure and predicted P. tricornutum UROD and CPOX homodimer/heterodimer. (A) UROD structure from Bacilius subtilis (PDB id:2INF) and Alphafold2.0 predicted P. tricornutum UROD homodimer and heterodimer. (B) CPOX structure from Acinetobacter baumannii (PDB id:5EO6) and Alphafold2.0 predicted putative P. tricornutum CPOX homodimer and heterodimer. The colour for the crystal structure represents experimental support confidence and the colour for the alphafold 2 prediction represents per-residue Local Distance Difference Test (pLDDT) confidence.
